# Discovery of Platyhelminth-Specific α/β-Integrin Families and Evidence for Their Role in Reproduction in *Schistosoma mansoni*

**DOI:** 10.1371/journal.pone.0052519

**Published:** 2012-12-27

**Authors:** Svenja Beckmann, Thomas Quack, Colette Dissous, Katia Cailliau, Gabriele Lang, Christoph G. Grevelding

**Affiliations:** 1 Justus-Liebig-University, Institute for Parasitology, Giessen, Germany; 2 University Lille, CIIL – Center for Infection and Immunity of Lille, Inserm U1019, Lille, France; 3 University Lille 1, Laboratoire de Régulation des Signaux de Division, Lille, France; Queensland Institute of Medical Research, Australia

## Abstract

In all metazoa, the response of cells to molecular stimuli from their environment represents a fundamental principle of regulatory processes controlling cell growth and differentiation. Among the membrane-linked receptors mediating extracellular communication processes are integrin receptors. Besides managing adhesion to the extracellular matrix or to other cells, they arrange information flow into the cells by activating intracellular signaling pathways often acting synergistically through cooperation with growth factor receptors. Although a wealth of information exists on integrins in different model organisms, there is a big gap of knowledge for platyhelminths. Here we report on the *in silico* detection and reconstruction of α and β integrins from free-living and parasitic platyhelminths, which according to structural and phylogenetic analyses form specific clades separate from each other and from further metazoan integrins. As representative orthologs of parasitic platyhelminths we have cloned one beta-integrin (Smβ-Int1) and four alpha-integrins (Smα-Int1 - Smα-Int4) from *Schistosoma mansoni*; they were characterized by molecular and biochemical analyses. Evidence is provided that Smβ-Int1 interacts and co-localizes in the reproductive organs with known schistosome cellular tyrosine kinases (CTKs), of which the Syk kinase SmTK4 appeared to be the strongest interaction partner as shown by yeast two-hybrid analyses and coimmunoprecipitation experiments. By a novel RNAi approach with adult schistosomes *in vitro* we demonstrate for the first time multinucleated oocytes in treated females, indicating a decisive role Smβ-Int1 during oogenesis as phenotypically analyzed by confocal laser scanning microscopy (CLSM). Our findings provide a first comprehensive overview about platyhelminth integrins, of which the parasite group exhibits unique features allowing a clear distinction from the free-living groups. Furthermore, we shed first lights on the functions of integrins in a trematode model parasite, revealing the complexity of molecular processes involved in its reproductive biology, which may be representative for other platyhelminths.

## Introduction

Integrins are non-covalently linked heterodimeric transmembrane receptors, present on most eukaryotic cells. They integrate signals provided by the extracellular matrix with intracellular signaling pathways acting in both directions (inside-out signaling and outside-in) across the plasma membrane [1]. Inside-out signaling is induced by conformational changes mediated by the cytoplasmic and transmembrane domains of integrins [2]–[3]. This subsequently influences ligand-binding affinities of the extracellular domains which, among others, regulates adhesion, cytoskeletal reorganization, cell proliferation, differentiation, or apoptosis as outside-in signaling processes [4]–[9]. Within metazoans integrins exhibit group-specific evolutionary differences in structure and subunit composition. Primitive bilateria have two integrin αβ-heterodimers, in *Caenorhabditis elegans* formed by one β- and two α-subunits [6]. Vertebrates have an expanded set of integrins, e.g. mammals have 8 β- and 18 α-subunits, known to assemble 24 distinct integrins [4], [6]. The extracellular domains of integrins recognize short peptide motifs as ligands, whose binding is specified by both subunits of a given αβ-heterodimer. The intracellular domains of integrins are small and comprise no intrinsic catalytic activity. Thus signals are transmitted through direct or indirect associations of the intracellular domains with other signaling molecules [6], [10], [11]. Among these are cellular protein tyrosine kinases (CTKs) such as Src and Syk kinases, which have already been shown to play significant roles during outside-in signaling processes [8]. While the primary function of integrins was previously assumed to be limited to adhesion processes, new evidence suggest integrins to synergistically cooperate with other molecules such as receptor tyrosine kinases (RTKs) in complex signaling pathways regulating growth and differentiation processes [4], [9], [12].

Due to their significant medical role, schistosomes are intensively studied parasites reaching the stage of model trematodes today. Second only to malaria, these blood flukes cause one of the most prevalent parasitic diseases for humans worldwide, called schistosomiasis. This infectious disease affects more than 200 million people but also animals [13]–[15]. The pathological consequences of an infection are caused by the eggs, which induce inflammatory reactions in the gut, bladder, spleen, and liver leading to granuloma formation and liver cirrhosis, the main cause of death of patients [16].

Schistosomes are the only trematodes living dioeciously. A nearly unique aspect of their biology, however, is the pairing-dependent induction and maintenance of the sexual maturation of the female. During a constant pairing contact, the male induces developmental processes in the female leading to the proliferation and differentiation of cells in the reproductive organs, such as the ovary and vitellarium [17]–[22]. This is a prerequisite for egg production leading to pathogenesis since only half of the ∼300 eggs produced daily reach the external environment by faeces or urine to continue life-cycling. The other half remains inside the host body being trapped in different organs. As typical for trematodes, schistosomes produce composite eggs, which are composed of one fertilized oocyte, originating from the ovary, and 30–40 vitelline cells produced in the vitellarium. Although the unique reproductive biology of schistosomes is long known, its molecular basis is still not understood. Not until the last years, first evidence has accumulated that signal transduction pathways control growth and differentiation of oocytes and vitellocytes following pairing. Several genes coding for signaling transduction proteins have been identified and characterized in *S. mansoni* with proven functions for their roles in mitogenic and differentiation processes in reproductive organs [23]–[25]. Among these were CTK members of the Src (SmTK3) [26], Src/Abl (SmTK6) [27]; Abl (SmAbl1/2) [28], and Syk families (SmTK4) [29]–[30]. Recently, it was demonstrated that SmTK6, SmTK4, and SmTK3 presumably act in a multi-kinase complex. First attempts to identify receptors, which may be able to activate this kinase complex identified SmVKR1, an atypical RTK of *S. mansoni* [27], [31]. However, evidence from other biological systems suggested that additional receptors may be involved such as integrins [8], [32]–[36].

Aims of our study were to investigate whether integrins exist in platyhelminths and to provide first evidence for their function in schistosomes as model trematodes. Towards this end we searched and reconstructed flatworm integrins *in silico* and performed comprehensive phylogenetic comparisons. In addition we have identified, cloned and sequenced one β-integrin (Smβ-Int1) and four α-integrins (Smα-Int1-4) of *S. mansoni*, which were used to complement the evolutionary analysis. Furthermore, we studied the tissue-specific transcriptional profiles of the schistosome integrins, and examined the potential of Smβ-Int1 to interact with known CTKs of *S. mansoni*. Finally, a novel RNAi approach was established to elucidate the role of Smβ-Int1. Data are presented demonstrating (i) the existence of four integrins in a free-living platyhelminth and five integrins in parasitic platyhelminths such as schistosomes that were characterized, (ii) the discovery of new platyhelminth-specific integrin clades containing branches discriminating orthologs of free-living and parasitic classes, (iii) co-localization of Smβ-Int1 with schistosome CTKs in the gonads, (iv) strong interaction of Smβ-Int1 with the schistosome Syk kinase SmTK4, and (v) the applicability of a novel combinatory siRNA approach which provided first evidence for a decisive role of Smβ-Int1 during oogenesis.

## Results

### Cloning and Sequence Analyses of Integrin Receptors from *S. mansoni*

**Figure 1:**
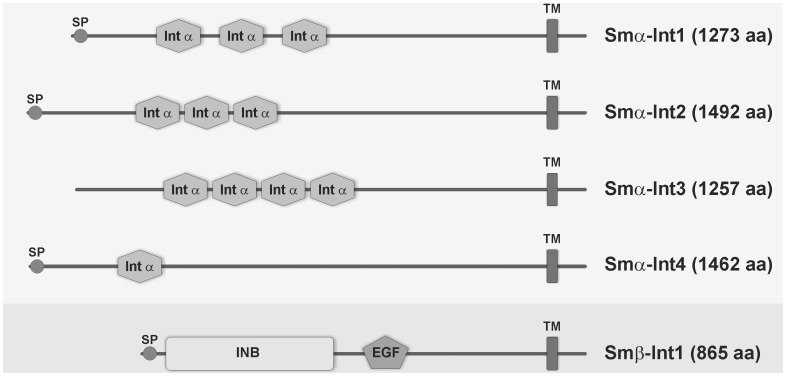
Domain structures of *S. mansoni* integrin receptors. Schematic structure of the schistosome α- and β-integrin receptors. Amino acid positions of the predicted conserved domains: Smα-Int1: 1273 aa; Int α 255–334, 359–418, 449–496; TM 1212–1234; Smα-Int2: 1492 aa; Int α 404–472, 476–546, 552–603; SP 1–30, 1296–1318; Smα-Int3: 1091 aa; Int α 257–316, 345–399; TM 980–1008; Smα-Int4: 1411 aa; SP 1–36; Int α 419–477; TM 1292–1314; Smβ-Int1: 865 aa; INB 36–470; EGF 567–604; TM 794–816;. (EGF: epidermal growth factor-like domain, Int α: integrin α domain (beta-propeller repeats), INB: integrin β domain, SP: signal peptide, TM: transmembrane domain).

Due to their high conservation in nature we expected to identify integrins in the *S. mansoni* genome data set [37]. Seven predicted gene sequences were identified encoding partial α-integrins as well as one predicted gene sequence encoding a β-integrin. The corresponding cDNAs were amplified in overlapping fragments, cloned, sequenced, and manually reconstructed from the overlaps where necessary (Fig. 1). The cDNA of the first α-integrin, Smα-Int1 (Smp_126140, chromosome 1; accession number FR749887), has a length of 3822 bp coding for a protein of 865 amino acids (aa). Smα-Int1 possesses a long N-terminal (N-term), extracellular domain with three integrin α domains (Int α, aa 255–334, 359–418, and 449–496) as well as one transmembrane domain (TM, aa 1212–1234), and a short intracellular C-terminal domain (C-term). Blast analyses showed similarity of Smα-Int1 to integrin α-5 orthologs from the parasitic trematode *Clonorchis sinensis* (accession number GAA56616.1, e = 0, 97% coverage, max. identity 53%), *Xenopus laevis* (accession number NP_001081072.1, 4e^−31^_,_ 58% coverage, max. identity 24%), *Homo sapiens* (accession number NP_002196.2; 7e^−30^_,_ 52% coverage, max. identity 24%), *Mus musculus* (accession number CAA55638.1, 4e^−30^_,_ 52% coverage, max. identity 25%), and *Rattus norvegicus* (accession number NP_001101588.1; 1e^−29^_,_ 52% coverage, max. identity 25%). In contrast to the human integrin α-5, Smα-Int1 possesses only three instead of five integrin α domains. The integrin α-5 from *C. sinensis* [38] has a similar length to Smα-Int1 (1329 aa), but exhibits five integrin α domains (aa 53–121, 221–279, 365–470, 490–549, and 576–623) like the human α-5 (Table S1 A). The cDNA of Smα-Int2 (Smp_170280, chromosome 1; accession number FR749888) is 4479 bp long and codes for a protein of 1492 aa. It contains an N-terminal signal peptide (aa 1–30) and a C-terminal transmembrane domain (aa 1296–1318). The big extracellular N-term of the molecule contains three integrin α domains (aa 404–472, 476–546, and 552–603), like Smα-Int1. Smα-Int2 exhibits similarity to the integrin α-ps from *C. sinensis* (accession number GAA54095.1, 4^−79^, 52% coverage, max. indentity 34%), but also to integrin α-2b from *X. laevis* (accession number NP_001088223.1, 6e^−11^, 10% coverage, max. identity 34%), *H. sapiens* (accession number EAW51596.1, 6e^−09^_,_ 12% coverage, max. indentity 30%), and *M. musculus* (accession number EDL34136.1, 1e^−08^_,_ 10% coverage, max. indentity 30%). Whereas the latter receptors possess five integrin α domains and a transmembrane domain, Smα-Int2 has only three integrin α domains and a transmembrane domain. The integrin α-ps from *C. sinensis* (2013 aa, reconstructed from three partial sequences (accession numbers GAA49531, GAA49530, and GAA54095) [38] also possesses an N-terminal signal peptide (aa 1–21), a C-terminal transmembrane domain (aa 1771–1793), but five integrin α domains (aa 362–422, 431–489, 860–923, 928–986, and 1032–1090) (Table S1 A). The third schistosome α-integrin Smα-Int3 was reconstructed from three predicted ORFs (Smp_156620, accession number FR749889, Smp_156610, and Smp_158350; located next to each other on chromosome 4). Its cDNA has a length of 3774 bp coding for a protein of 1257 aa, which possesses four integrin α domains (aa 178–253, 257–316, 345–399, and 408–462) and a C-terminal transmembrane domain (aa 1152–1174). Blast analyses indicated that Smα-Int3 is homologous to integrin α-7 from *C. sinensis* (accession number GAA52225.1, e = 0, 87% coverage, max. indentity 51%), *H. sapiens* (accession number AAC18968.1; 2e^−34^_,_ 39% coverage, max. indentity 29%), *R. norvegicus* (accession number NP_110469.1; 8e^−35^, 38% coverage, max. indentity 29%), or *M. musculus* (accession number AAA16600.1, 1e^−34^, 39% coverage, max. indentity 28%). Although homology exists, there are differences since the human and rodent integrin α-7 receptors each possess five integrin α domains and a transmembrane domain. In comparison, integrin α-7 from *C. sinensis* contains four integrin α domains (aa 136–186, 396–457, 482–536, 545–593), but no transmembrane domain, which might be due to a partial gene prediction (Table S1 A). We identified two further sequences with homology to α-integrins (Smp_181010 and Smp_173540; both located next to each other on chromosome 2) in the genome of *S. mansoni*, and reconstructed from these sequences the fourth integrin α, Smα-Int4. Its cDNA has a predicted length of 4389 bp encoding a protein of 1462 aa, which possesses one predicted signal peptide (aa 1–36), one integrin α domain (aa 496–550), as well as one transmembrane domain (aa 1306–1328) (Table S1A). According to Blast analyses, Smα-Int4 is homologous to α-integrin from *C. sinensis* (accession number GAA28731.2, e = 0, 96% coverage, max. indentity 40%), and similar to the nematode parasites *Trichinella spiralis* (accession number XP_003378647.1, 2e^−07^, 32% coverage, max. indentity 29%) and *Loa loa* (accession number XP_003143418.1, 8e^−06^, 25% coverage, max. indentity 21%), the mosquito *Aedes aegypti* (accession number XP_001659751.1, 2e^−08^, 25% coverage, max. indentity 35%), but only distantly related to to vertebrate α-integrins like the α-8 receptor from *M. musculus* (accession number AAC15665.1, 0.027, 9% coverage, max. indentity 27%), which all possess more than one integrin α domain.

The cDNA of the identified β-integrin receptor Smβ-Int1 (Smp_089700, chromosome 1; accession number FR749886) has a length of 2598 bp coding for a protein of 2598 aa. It contains a long extracellular, N-terminal domain containing a signal peptide (aa 1–25), an integrin β domain (Int β, aa 36–470), and an epidermal growth factor-like domain (EGF, aa 567–604) followed by the transmembrane domain (aa 794–816), and a short intracellular C-terminus without conserved domains (Fig. 1). Within the Integrin β domain conserved PSI (aa 30–78) and VWA domains (aa 135–392) occur (Table S1 B). The N-terminal PSI domain is a Cys-rich region with homology to membrane proteins such as plexins, semaphorins, and the c-met receptor (termed for plexins, semaphorins and integrins) [39]. Seven Cys residues occur in β-integrin PSI domains of higher eukaryotes [40], from which six are also present within the PSI domain of Smβ-Int1. The first of these cysteines is described to form a long-range S-S bond to the C-terminal cysteine-rich region, which is N-terminal of the EGF-like domain [41] and holds the integrin in the inactive conformation [42]. In Smβ-Int1 such a S-S bond is possible between the cysteine residues at positions 31 (first cysteine residue in the PSI domain) and position 563 (last Cys residue N-terminal of the EGF-like domain). The VWA domain (von Willebrand factor type A domain) is evolutionarily conserved and spans approximately 100–340 aa, 240 aa in β-integrins of higher eukaryotes [40]. The VWA domain in Smβ-Int1 covers 257 aa (aa 135–392). This domain is the major ligand-binding site, containing a putative metal-binding DXSXS sequence motif [40], [43], which is located at position 143–147 in Smβ-Int1 differing in the last residue, where a Thr instead of a Ser is located (DLSYT). Beside these domains Smβ-Int1 possesses a EGF-like domain (aa 567–604), containing a high number of Cys residues like other eukaryotic β-integrins at similar positions (aa 435–600) [40]. According to Blast analyses, Smβ-Int1 showed homology to the integrin β-1 from *C. sinensis* (accession number GAA31131.2, e = 0, 88% coverage, max. identity 78%), but also to integrin β-2 from *H. sapiens* (accession number NP_000202.2, 3e^−139^_,_ 96% coverage, 33% max. identity), *X. laevis* (accession number NP_001080017.1, 8e^−142^_,_ 98% coverage, 33% max. identity), Integrin β-1 from *M. musculus* (accession number NP_034708.1, 4e^−135^, 95% coverage, 34% max. identity), *R. norvegicus* (accession number NP_058178.2, 7e^−134^_,_ 95% coverage, 34% max. identity), *Gallus gallus* (accession number NP_001034343.2, 5e^−133^_,_ 98% coverage, 33% max. identity), and to Integrin β-7 from *H. sapiens* (accession number NP_000880.1, 1e^−131^_,_ 96% coverage, 33% max. identity).

### *In silico* Identification and Reconstruction of Integrins from other Parasitic or Free-living Worms Uncovered Novel, Platyhelminth-specific Integrin Families

**Figure 2:**
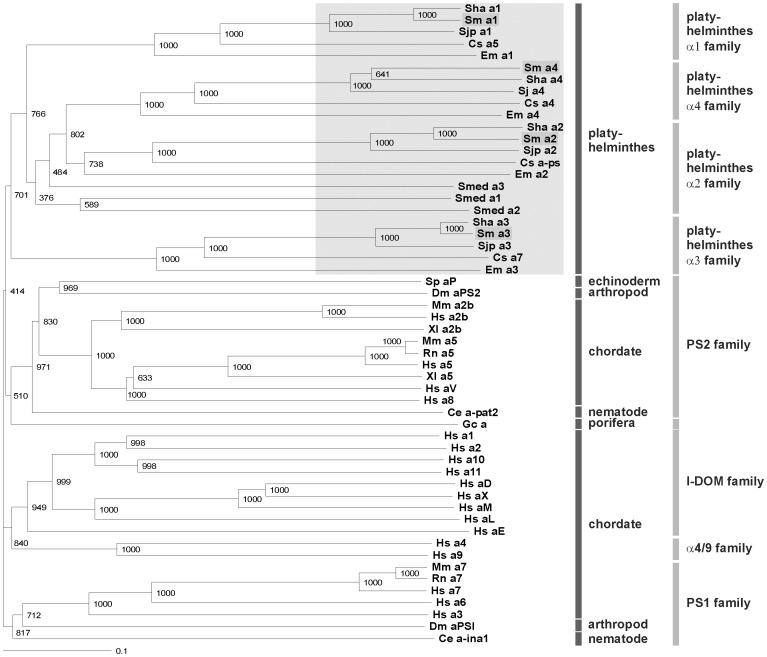
Phylogenetic analyses showing the unique status of plathyhelminth α-integrins. Phylogram of the analysis of the full-length sequences of the *S. mansoni* α-integrin receptors Smα-Int1, Smα-Int2, Smα-Int3, and other α-integrin receptors using CLUSTAL X (www.clustal.org) and TreeViewX. The phylogenetic relationship was deduced using the Bootstrap Neighbour-Joining (N–J) method and the bootstrap values were generated based on 1000 bootstrap trails with a random number generator seed of 100. Sequences were obtained from the National Centre for Biotechnology Information using the WWW Entrez Browser (www.ncbi.nlm.nih.gov), Swiss-Prot (www.uniprot.org), GeneDB (www.genedb.org), and the *Schmidtea mediterranea* Genome Database (http://smedgd.neuro.utah.edu/). The corresponding protein numbers are: Sha a1 (α-integrin 1 receptor, *S. haematobium*; Sha_102401), Sm a1 (α-integrin 1 receptor, *S. mansoni*; FR749887), Sjp a1 (α-integrin 1 receptor, *S. japonicum*; Sjp_0037690), Cs a5 (α-integrin 5 receptor, *Clonorchis sinesis*; GAA56616.1), Em a1 (α-integrin 1 receptor, *Echinococcus multilocularis*; EmuJ_000215000 ), Sm a4 (α-integrin 4 receptor, *S. mansoni*; Smp_1735401, Smp_181010), Sha a4 (α-integrin 4 receptor, *S. haematobium*; Sha_104436, Sha_106831), Sjp a4 (α-integrin 4 receptor, *S. japonicum*; Sjp_0046780, Sjp_0046790), Cs a4 (α-integrin 4 receptor, *Clonorchis sinesis*; GAA28731), Em a4 (α-integrin 4 receptor, *Echinococcus multilocularis*; EmuJ_000573500), Sha a2 (α-integrin 2 receptor, *S. haematobium*; Sha_106921), Sm a2 (α-integrin 2 receptor, *S. mansoni*; FR749888), Sjp a2 (α-integrin 2 receptor, *S. japonicum*; Sjp_0069490), Cs a-ps (α-integrin-ps receptor, *Clonorchis sinesis*; GAA54095, GAA49531, GAA49530), Em a2 (α-integrin 2 receptor, *Echinococcus multilocularis*; EmuJ_000192500 ), Smed a3 (α-integrin 3 receptor, *Schmidtea mediterranea*; lcl|mk4.000046.14.01), Smed a1 (α-integrin 1 receptor, *Schmidtea mediterranea*; lcl|mk4.001411.00.01), Smed a2 (α-integrin 2 receptor, *Schmidtea mediterranea*; lcl|mk4.003797. 00.01), Sha a3 (α-integrin 3 receptor, *S. haematobium*; Sha_102914), Sm a3 (α-integrin 3 receptor, *S. mansoni*; FR749889, Smp_156610, Smp_156620), Sjp a3 (α-integrin 3 receptor, *S. japonicum*; Sjp_0063430, Sjp_0063420), Cs a7 (α-integrin 7 receptor, *Clonorchis sinesis*; GAA52225.1), Em a3 (α-integrin 3 receptor, *Echinococcus multilocularis*; EmuJ_000782500), Sp aP (α-integrin P receptor, *Strongylocentrotus purpuratus*, AF177914), Dm aPS2 (α-integrin PS2 receptor, *Drosophila melanogaster*, Q24247), Mm a2b (α-integrin 2b receptor, *Mus musculus*; EDL34136.1), Hs a2b (α-integrin 2b receptor, *Homo sapiens*; EAW51595.1), Xl a2b (α-integrin 2b receptor, *Xenopus laevis*; NP_001088223.1), Mm a5 (α-integrin 5 receptor, *Mus musculus*; CAA55638.1), Rn a5 (α-integrin 5 receptor, *Rattus norvegicus*; NP_001101588.1), Hs a5 (α-integrin 5 receptor, *Homo sapiens*; NP_002196.2), Xl a5 (α-integrin 5 receptor, *Xenopus laevis*; NP_001081072.1), Hs aV (α-integrin V receptor, *Homo sapiens*; P06756), Hs a8 (α-integrin 8 receptor, *Homo sapiens*; P53708), Ce a-pat2 (α-integrin pat-2, *Ceanorhabditis elegans*; P34446), Gc a (α-integrin receptor, *Geodia cydonium*; X97283), Hs a1 (α-integrin 1 receptor, *Homo sapiens*; P56199), Hs a2 (α-integrin 2 receptor, *Homo sapiens*; P17301), Hs a10 (α-integrin 10 receptor, *Homo sapiens*; O75578), Hs a11 (α-integrin 11 receptor, *Homo sapiens*; Q9UKX5), Hs aD (α-integrin D receptor, *Homo sapiens*; Q13349), Hs aX (α-integrin X receptor, *Homo sapiens*; P20702), Hs aM (α-integrin M receptor, *Homo sapiens*; P11215), Hs aL (α-integrin L receptor, *Homo sapiens*; P20701), Hs aE (α-integrin E receptor, *Homo sapiens*; P38579), Hs a4 (α-integrin 4 receptor, *Homo sapiens*; P13612), Hs a9 (α-integrin 9 receptor, *Homo sapiens*; Q13797), Mm a7 (α-integrin 7 receptor, *Mus musculus*; AAA16600.1), Rn a7 (α-integrin 7 receptor, *Rattus norvegicus*; NP_110469.1), Hs a7 (α-integrin 7 receptor, *Homo sapiens*; EAW96822.1), Hs a6 (α-integrin 6 receptor, *Homo sapiens*; P23229), Hs a3 (α-integrin 3 receptor, *Homo sapiens*; P26006), Dm aPSI (α-integrin PSI receptor, *Drosophila melanogaster*, Q24247), and Ce a-ina1 (α-integrin ina1, *Ceanorhabditis elegans*; Q03600).

**Figure 3:**
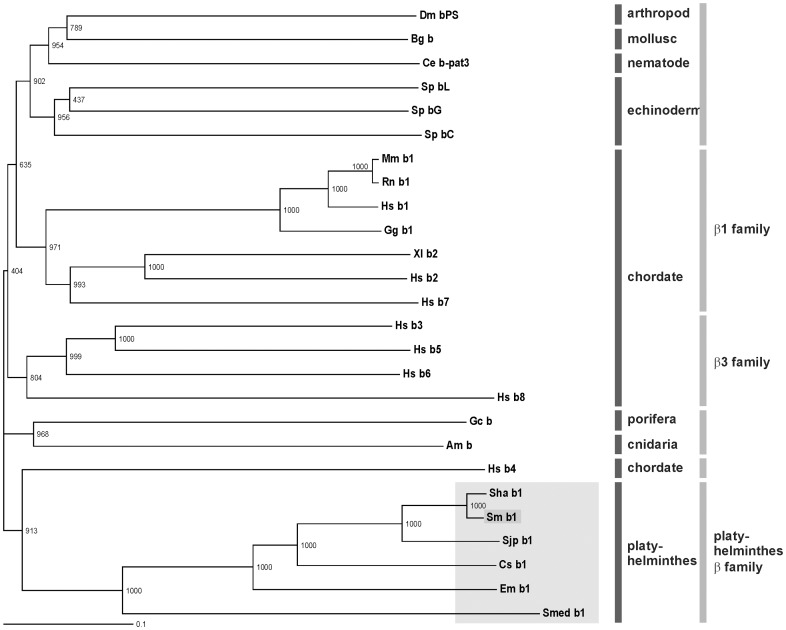
Phylogenetic analyses showing the unique status of plathyhelminth β-integrins. Phylogram of the analysis of the full-length sequences of the *S. mansoni* β-integrin receptor Smβ-Int1 and other β-integrin receptors using CLUSTAL X (www.clustal.org) and TreeViewX. The phylogenetic relationship was deduced using the Bootstrap Neighbour-Joining (N–J) method and the bootstrap values were generated based on 1000 bootstrap trails with a random number generator seed of 100. Sequences were obtained from the National Centre for Biotechnology Information using the WWW Entrez Browser (www.ncbi.nlm.nih.gov), Swiss-Prot (www.uniprot.org), GeneDB (www.genedb.org), and the *Schmidtea mediterranea* Genome Database (http://smedgd.neuro.utah.edu/). The corresponding protein accession numbers are: Dm bPS (β-integrin PS, *Drosophila melanogaster*; P11584), Bg b (β-integrin, *Biomphalaria glabrata*; AF060203), Ce b-pat3 (β-integrin pat-3, *Ceanorhabditis elegans*; Q27874), Sp bL (β-integrin L subunit, *Strongylocentrotus purpuratus*; NP_999731), Sp bG (β-integrin G subunit, *Strongylocentrotus purpuratus*; NP_999732), Sp bC (β-integrin C subunit, *Strongylocentrotus purpuratus*; AF0559607), Mm b1 (β-integrin 1 receptor, *Mus musculus*; NP_034708.1), Rn b1 (β-integrin 1 receptor, *Rattus norvegicus*; NP_058718.2), Hs b1 (β-integrin 1 receptor, *Homo sapiens*; P05556), Gg b1 (β-integrin 1 receptor, *Gallus gallus*; NP_001034343.2), Xl b2 (β-integrin 2 receptor, *Xenopus laevis*; NP_001080017.1), Hs b2 (β-integrin 2 receptor, *Homo sapiens*; NP_000202.2), Hs b7 (β-integrin 7 receptor, *Homo sapiens*; NP_000880.1), HS b3 (β-integrin 3 receptor, *Homo sapiens*; P05106), Hs b5 (β-integrin 5 receptor, *Homo sapiens*; P18084), Hs b6 (β-integrin 6 receptor, *Homo sapiens*; P18564), Hs b 8 (β-integrin 8 receptor, *Homo sapiens*; P26012), Gc b (β-integrin receptor, *Geodia cydonium*; O97189), Am b (β-integrin, *Acropora millepora*; AF005356), Hs b4 (β-integrin 4 receptor, *Homo sapiens*; P16144), Sha b1 (β-integrin 1 receptor, *S. haematobium*; Sha_105750), Sm b1 (β-integrin 1 receptor, *S. mansoni*; FR749886), Sjp b1 (β-integrin 1 receptor, *S. japonicum*; Sjp_0081260), Cs b1 (β-integrin 1 receptor, *Clonorchis sinesis*; GAA31131.2), Em b1 (β-integrin 1 receptor, *Echinococcus multilocularis*; EmuJ_000528400), and Smed b1 (β-integrin 1 receptor, *Schmidtea mediterranea*; lcl|mk4.001280.01.01).

In a more detailed data base analysis we searched for and reconstructed sequences of α- and β-integrin receptors in the genome data sets of the related schistosome species *Schistosoma haematobium* [44], *Schistosoma japonicum* [45], or other parasitic platyhelminthes such as the trematode *C. sinensis* [38], the cestode *Echinococcus multilocularis* (http://www.genedb.org/Homepage/Emultilocularis), as well as the free-living planarian *Schmidtea mediterranea* [46] (Table S1). Only partial sequences of α-Int1/2/3 and no α-Int4 sequence were found in the *S. mediterranea* genome data set. Within the *S. haematobium* genome data only a partial α-Int4 sequence could be identified. Phylogenetic analyses of the four schistosome α integrins with different other vertebrate and invertebrate α integrins demonstrated that the schistosome homologs clustered together with orthologs from *S. haematobium*, *S. japonicum*, *C. sinensis*, *E. multilocularis,* as well as with *S. mediterranea* forming specific clades (Fig. 2). The trematodes α integrins showed no further clustering with common α integrin families like the PS1, PS2, I-DOM, or α4/9 [47]. Inside this novel group of platyhelminth-specific α-integrins, and in accordance with the presence of three to four α-integrins in each species, the receptors clustered in four families, which we called platyhelminth α1-, α2-, α3-, and α4-families. In a phylogenetic analysis with different integrin β receptors, the *S. mansoni* homolog clustered with orthologs from *S. haematobium, S. japonicum, C. sinensis, E. multilocularis,* and *S. mediterranea*. The only non-platyhelminth integrin receptor clustering with this group was the human integrin β4 (Fig. 3). Interestingly, it was previously shown that this receptor is the only vertebrate integrin ortholog clustering outside of the β1 and β3 integrin families [47]. Our phylogenetic analysis suggests that human β4 may have an evolutionary ancient origin, clustering in an own group close to the platyhelminth orthologs. Since this group was also found to be unique, it was named platyhelminth β-family. Within the platyhelminth α-integrin families and the platyhelminth β-family the phylogenetic relationship of platyhelminthes is perfectly reflected [44].

### *In situ* Hybridizations Demonstrated Integrin Transcription in Gonad Tissues

**Figure 4:**
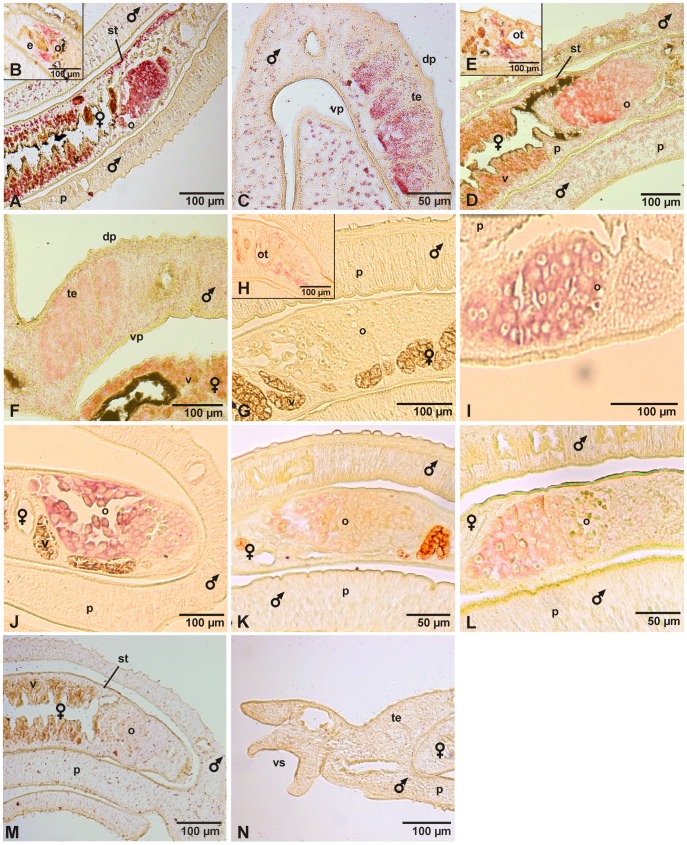
*In situ*-hybridization localized transcripts of Smβ-Int1, Smα-Int1- Smα-Int4 in the gonads and gonad-associated tissues of adult *S. mansoni*. Representative sections (5 µm) of adult schistosome couples are shown (males and females are indicated), which were hybridized with DIG-labeled antisense-RNA probes of Smβ-Int1 (A–C), Smα-Int1 (D–F), Smα-Int2 (G, H), Smα-Int3 (I), Smα-Int4 (K) and for control with a DIG-labeled sense-RNA probe of Smα-Int3 (J), Smα-Int4 (L), or Smβ-Int1 (M, N). mRNA transcripts of Smβ-Int1 were detected in the ovary (o), the ootype-surrounding area (ot) anterior the ovary, the vitellarium (v), the subtegument (st), the testes (te), and the parenchyma (p) of both genders. Transcripts of Smα-Int1 were also detected in the ovary (o), the ootype-surrounding area (ot), and the vitellarium (v) of the female, the testes of the male, and the parenchyma (p) of both genders. Transcripts of Smα-Int2 were exclusively detected in the ootype-surrounding area (ot) anterior the ovary (o). Antisense and sense transcripts of Smα-Int3 and Smα-Int4 were only detected in the ovary (o). No signals were detected using sense transcripts of Smβ-Int1 (K, L), Smα-Int1, and Smα-Int2 (unpublished). dp: dorsal part; vp: ventral part; vs: ventral sucker; scale bars as indicated.

RT-PCR analyses showed that the schistosome α- and β-integrins are transcribed in larval (miracidia, but not in cercariae) and adult life stages (data not shown). To investigate their tissue-specific expression *in situ*-hybridizations were performed. Transcripts of Smβ-Int1 were localized predominantly in the ovary, the vitellarium (Fig. 4A), and the ootype-surrounding area (Fig. 4B), as well as in some areas of the subtegument of the female (Fig. 4A), the testes of the male (Fig. 4C), and some cells of the parenchyma of both genders (Fig. 4A, C). Within the testes, the detected signals were stronger in the ventral part of the testicular lobes. Since in this area the mature elongated sperms are located [23], [48], these results indicated stronger transcriptional activity of Smβ-Int1 in mature spermatozoa than in immature spermatocytes. A similar tissue-specific pattern was observed for Smα-Int1. Transcripts were predominantly detected in the ovary, the vitellarium (Fig. 4D), and the ootype-surrounding area (Fig. 4E) of the female, in the testes of the male (Fig. 4F), and in the parenchyma of both genders (Fig. 4D, F). Transcripts of Smα-Int2 were exclusively detected in the ootype-surrounding area anterior the ovary, but not within the ovary (Fig. 4G, H). No signals were detected using sense probes of Smβ-Int1 (Fig. 4M, N), Smα-Int1, or Smα-Int2 (not shown) indicating that antisense regulation may not occur for these integrins. However, *in situ* hybridizations with probes specific for Smα-Int3 or Smα-Int4 led to different results. Antisense as well as sense transcripts of Smα-Int3 as well as Smα-Int4 (Fig. 4I–L) were detected in the ovary indicating that the expression of these two α-integrins might be post-transcriptionally regulated by antisense RNA, a phenomenon increasingly reported for some schistosome genes including those involved in signal transduction [49]–[50].

### Interaction Studies in the Yeast Two-hybrid System and Co-immunoprecipitation Confirmed Smβ-Int1-CTK Interactions

**Figure 5:**
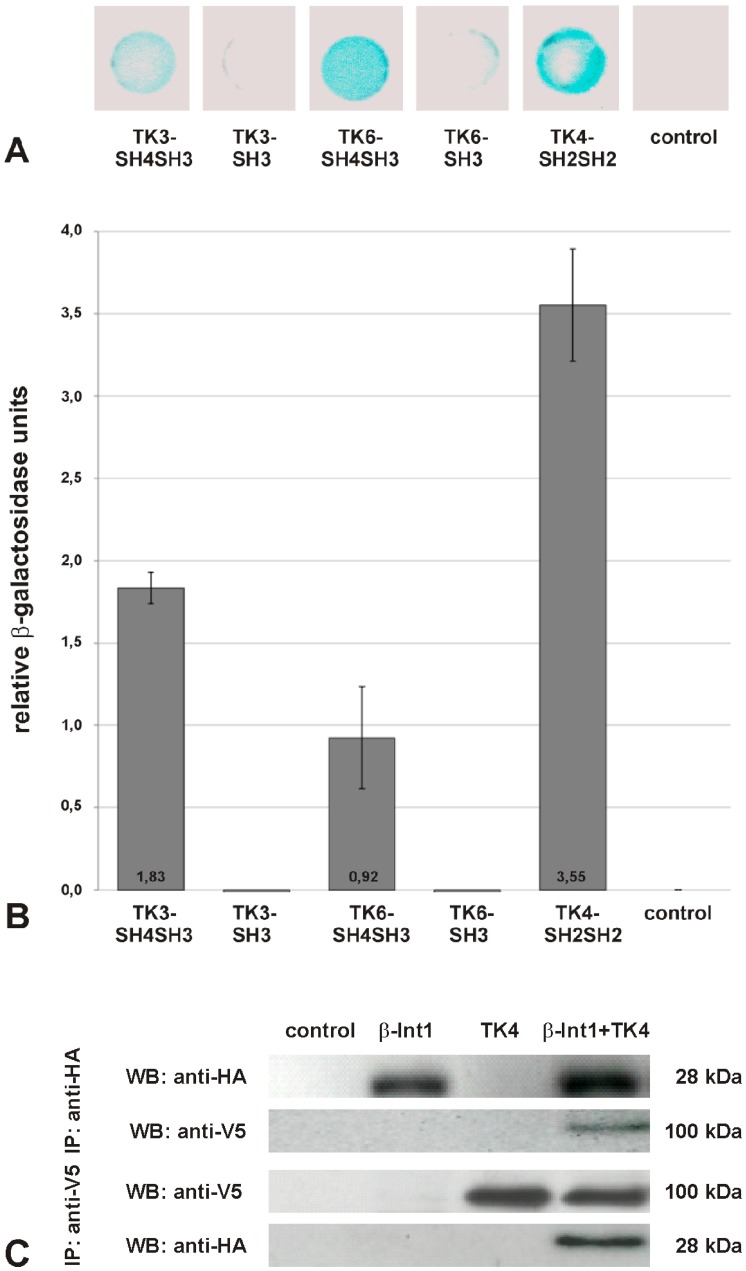
Interaction studies confirmed binding of Smβ-Int1 to the schistosome cellular kinases SmTK3, SmTK6, and SmTK4. A: For binding studies in the YTH system, yeast cells (strain AH109) were co-transformed with the prey plasmid Smβ-Int1-C-term pACT2 together with the baits SmTK3-SH4SH3 pBridge, SmTK3-SH3 pBridge, SmTK6-SH4SH3 pBridge, SmTK6-SH3 pBridge, and SmTK4-SH2SH2 pBridge. Yeast clones were selected on Trp^−^/Leu^−^/His^−^ media (T^−^/L^−^/H^−^) for interactions between bait and prey proteins, and β-galactosidase colony lift filter assays were performed for detection of LacZ expression. **B:** Comparative β-galactosidase liquid assays were performed with the yeast clones from A to determine the relative binding strengths. As control, untransformed yeast cells (AH109) were used (control). The statistical evaluation of seven independent measurements of β-Gal activity (n = 7) is shown (error bars are indicated). **C:** Co-Immunoprecipitation of HA-Smβ-Int1 and V5-SmTK4 expressed in *Xenopus* oocytes. Anti-HA antibodies immunoprecipitated Smβ-Int1 together with SmTK4 upon co-expression in oocytes. Inversely, anti-V5 antibodies immunoprecipitated SmTK4 with Smβ-Int1, when they were expressed together in oocytes.

Integrins are discussed to transduce signals via their intracellular part to signaling molecules such as Src and Syk CTKs [7], [32], [51]. Since evidence has been obtained for a Src-Syk kinase complex in the reproductive organs of schistosomes [23], [24], [27], we performed interaction studies in the YTH system to investigate the potential of Smβ-Int1 to be involved as an upstream partner. To this end the intracellular C-term of Smβ-Int1 was expressed in yeast as a fusion protein with the Gal4-AD, and also the N-term protein interaction parts of schistosome CTKs (SH4 and/or SH3 domains of the Src kinases SmTK3 and SmTK6; tandem SH2 domain of the Syk kinase SmTK4) were co-expressed as fusion proteins with the Gal4-BD. All yeast clones survived growth selection (Trp^−^/Leu^−^/His^−^), but β-Gal colony lift filter-assays showed LacZ expression exclusively for yeast clones which were transformed with Smβ-Int1-C-term together with the SH4SH3 domains of SmTK3 and SmTK6, respectively, as well as with SmTK4-SH2SH2, indicating interactions of all three CTKs with the intracellular part of Smβ-Int1 (Fig. 5A). To quantify and compare the strengths of interactions, β-Gal liquid assays were performed. In all cases the interactions were confirmed, although the strongest interaction occurred between the SmTK4 SH2SH2-domain and Smβ-Int1 (Fig. 5B).

To confirm the strong interaction between Smβ-Int1 and SmTK4, a co-immunoprecipitation experiment was performed. To this end, we co-expressed in *Xenopus* oocytes the HA-tagged intracellular part of Smβ-Int1 and the V5-tagged SmTK4 kinase. Oocyte lysate proteins were immunoprecipitated with each anti-tag antibody. The results showed the presence of V5-SmTK4 in the immune complexes isolated with anti-HA antibodies and, inversely, the presence of HA-Smβ-Int1 in the V5 immune complexes, thus confirming the co-immunoprecipitation of both proteins and their interactions (Fig. 5C).

### A Novel RNAi Approach Revealed a Multinucleated Oocyte Phenotype for Smβ-Int1 Deficiency

For functional analyses we selected Smβ-Int1 as target due to its unique role as universal dimerization partner of the schistosome α integrins, its interactions and co-localizations with the schistosome CTKs in gonad tissues. Additionally, we chose Smα-Int1 as target, since it showed similar localization as well as transcriptional regulation patterns. In analogy to previous inhibitor approaches [23], [29] we tried to inhibit Smβ-Int1 function by Echistatin, a viper disintegrin representing the most potent inhibitor of β-integrins [52]. Echistatin contains a cysteine-rich peptide containing the Arg-Gly-Asp (RGD) sequence, which simulates a ligand-binding motif thus acting as potent, irreversible antagonist for β-integrin [53]–[54]. Regarding cell culture experiments in mammalian cells Echistatin has an IC_50_ value of 0.1–30 nM [53]–[55].

Adult *S. mansoni* couples were treated for 5 days with 100 nM, or for 3 days with 500 nM Echistatin *in vitro*. During these time periods no changes in physiology or behavior were observed, and no morphological abnormalities compared to control couples were detected using confocal laser scanning microscopy (CLSM)(not shown).

As an additional possibility to affect integrin function we performed RNAi experiments according to recently established protocols [29], [56]. To this end schistosome couples were electroporated followed by soaking with dsRNAs specific for Smα-Int1 or Smβ-Int1. After 5 days, only slight reductions of the corresponding transcripts of 15–25% (Smα-Int1) or 10–20% (Smβ-Int1) were achieved compared to control worms electroporated without dsRNA. Morphological analyses by CLSM revealed no differences between dsRNA-treated versus control worms (unpublished).

**Figure 6:**
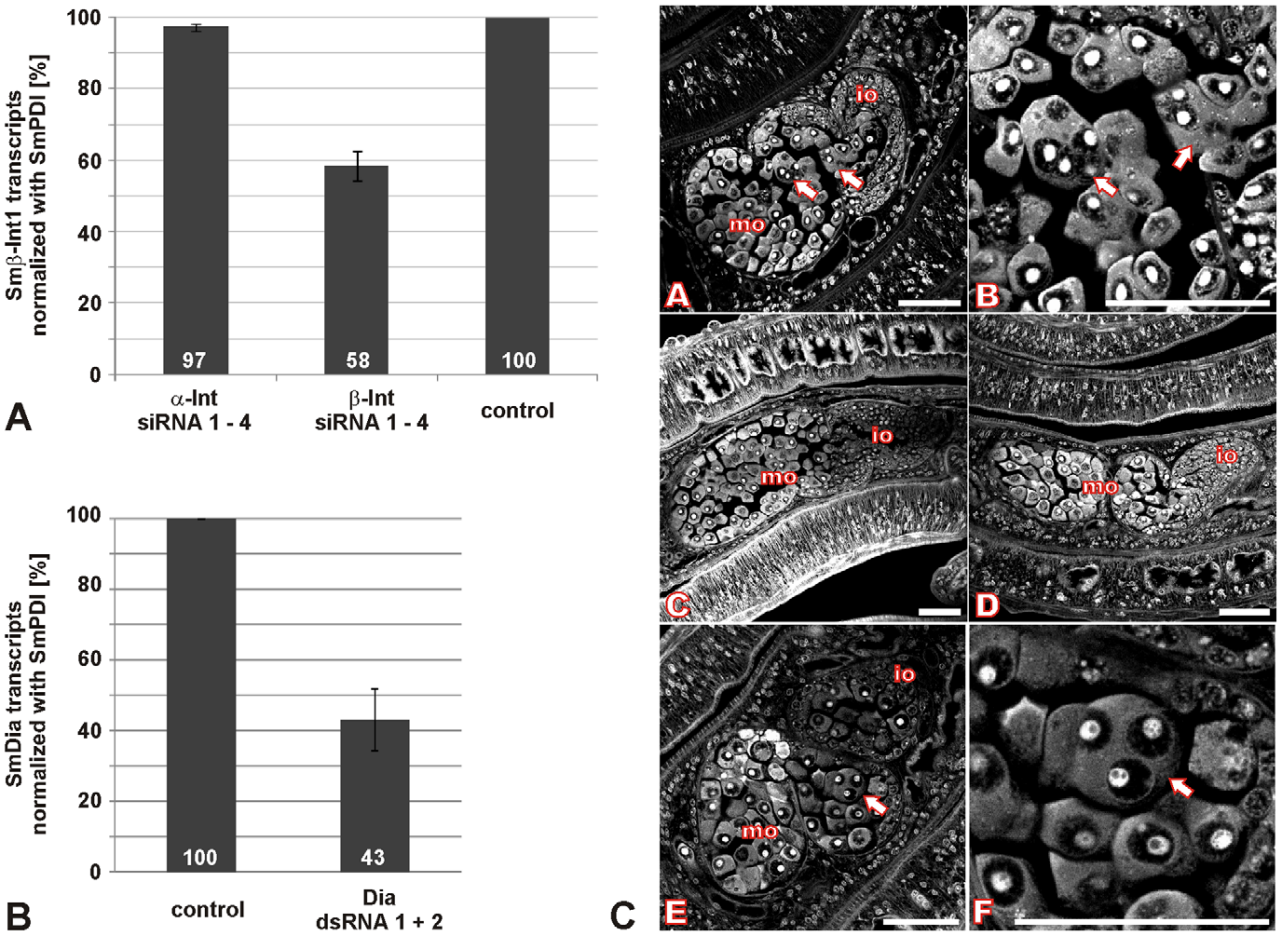
RNAi knocking-down Smβ-Int1 and SmDia led to morphological changes in the ovary of *S. mansoni* females. A: Using a combination of four siRNAs specific for Smβ-Int1 (β-Int1 siRNAs 1–4), a Smβ-Int1-transcript reduction down to 58% was determined by semi-quantitative RT-PCRs (n = 2) compared to control worms (no siRNAs), or worms electroporated with Smα-Int1-specific siRNAs (α-Int1 siRNA 1–4). **B:** The combination of two dsRNAs specific for SmDia led to a SmDia-transcript reduction down to 43% compared to control worms (no dsRNAs), which was determined by semi-quantitative RT-PCRs (n = 3). **C:** Confocal scanning laser microscope images of carmine red-stained whole-mount preparations of *S. mansoni* couples treated with siRNA specific for Smβ-Int1 (A, B), Smα-Int1 (C), without si/dsRNA (D), or with SmDia-specific dsRNAs (E, F). io: immature oocytes, mo: mature oocytes, arrows: poly-nucleated oocytes; scale bars 50 µm.

As an alternative we tested siRNAs as templates for the RNAi machinery in schistosomes. The siRNA sequences were predicted by a specific algorithm to find optimized target sequences, and a special sequence motif was added to the selected sequences increasing the efficiency of gene silencing (www.riboxx.com). Four different siRNAs were designed for each of the two schistosome integrins. Attempts with individual siRNAs (2.5 µg per electroporation) failed to significantly reduce the amount of transcripts of Smα-Int1 or Smβ-Int1. To overcome this limitation we combined all four siRNAs specific for Smβ-Int1 using 2.5 µg of each siRNA. This combinatory approach led to a reduction of the Smβ-Int1 transcript level down to 58% compared to transcript levels in control worms electroporated without siRNAs or Smα-Int1-specific siRNAs (Fig. 6A). A similar combinatorial approach failed to reduce the amount of Smα-Int1 transcripts (not shown). CLSM analyses of the couples electroporated with all four Smβ-Int1-siRNAs revealed morphological changes within the ovaries of most of the treated females (Fig. 6C–A, B). The number of big primary oocytes was slightly increased, and they were more distributed into the anterior part of the ovary (Fig. 6C–A), where in wildtype ovaries only immature oocytes are located (Fig. 6C–D) [23], [48]. In the posterior part of the ovary, abnormally shaped primary oocytes were detected. Closer inspection revealed the presence of multiple nuclei in these oocytes (Fig. 6C–A, B). In control females, all oocytes were roundly shaped containing a single nucleus, as expected (Fig. 6C, D). In the other organs of females or males, no morphological changes were observed. Worms electroporated with Smα-Int1-specific siRNAs showed no morphological differences within the ovary (Fig. 6C–C) or elsewhere compared to control worms (Fig. 6C–D).

## Discussion

Using the *S. mansoni* genome data set [37] we identified sequences coding for four potential α-integrins as well as for one β-integrin, which were cloned and sequenced. In the genome data sets of the schistosome species *S. haematobium* [44] and *S. japonicum* (*Schistosoma japonicum* Genome Sequencing and Functional Analysis Consortium, 2009), the closely related trematode *C. sinensis* [38], the cestode *E. multilocularis*, and the free-living planarian *S. mediterranea* [46] four α-integrin subunits (in *S. mediterranea* only three) and one β-integrin subunit could be also identified, which are similar in length and domain structures to the *S. mansoni* receptors. Smα-Int2 is the longest α-integrin and has, as Smα-Int1, a C-terminal transmembrane domain. As typical for α-integrins, the intracellular domains of Smα-Int1 and Smα-Int2 are short and lack conserved domains. Smα-Int3 is the smallest one containing also a transmembrane domain as Smα-Int4, which has only one α-integrin domain. Thus it may represent an archetype of this receptor class.

Human α-integrins were divided into five distinct functional clades - the laminin binding PSI, the RGD tri-peptide binding PS2, the α4/α9, and the I-DOM clade containing collagen binding as well as leukocyte-specific α-integrins [57]. The α-integrin subunit homologs from the model invertebrates *C. elegans* and *Drosophila melanogaster*, as well as from the sea urchin *Strongylocentrotus purpuratus,* clustered in the laminin clade (PS1) and the RGD binding (PS2) clade. In contrast, the monophyletic I-DOM clade was suggested to be a chordate invention [58]. An outgroup is formed by the α-integrin homolog of the sponge *Geodia cydonium* (Fig. 2). The α-integrins from platyhelminthes form a new clade that is divided into four sub-clades, where the α4 and α2 families are closer related to each other than to the α1 or α3 families. The α-integrins from the three schistosome species as well as from the liver fluke *C. sinensis* and the cestode *E. multilocularis* clustered together with 100% bootstrap support except Shaα-Int4 (64%) and Emα-Int2 (74%) leading to the hypothesis that the differentiation of the four α-integrin subtypes occurred before the evolution of the different parasitic platyhelminthes, but also after the divergence from the free-living ones. The α-integrins of the free-living planarian *S. mediterranea* clustered only with significant lower bootstrap support (38–48%; Fig. 2), branching separately from the parasite orthologs. Furthermore, planarians presumably possess only three α-integrin subunits.

The β-integrin family of vertebrates can be divided into two main branches, the β1 clade with the β-integrin ancestor β1, the β3 clade containing all β-integrin subunits specific to RGD ligand binding integrins. The β4 subunit is positioned separately to the other vertebrate clades, and may have separated from the other β-integrins prior to the origin of vertebrates [47]. The latter fact presumably explains the phylogenetic proximity to the platyhelminth β-integrins. Invertebrate β-integrins like those from e.g. *C. elegans*, *D. melanogaster*, *S. purpuratus, G. cydonium* do not cluster with vertebrate sequences [58]. With highly significant (100% of 1000 bootstrap pseudo-samples) bootstrap support the platyhelminth β-integrins form a new clade distinct from those of vertebrates, but also from the other invertebrate orthologs (Fig. 3). Already in cnidaria and porifera β-integrins were identified indicating that these proteins existed in the earliest stages of metazoan evolution, and it is supposed that they function also in these organisms not only as adhesion molecules, but fulfill additional roles during outside-in signaling [58]–[59]. In general, the structure of β-integrins from vertebrates and invertebrates is similar indicating comparable functions. Ligands of invertebrate integrins are not well characterized, but at least laminin- as well as ligands containing RGD-motifs were identified [60]. Within the extracellular domain of human β-integrins a defined amino acid motif occurs (DLSYSMKDDLWN) that is involved in RGD ligand binding, for which the first Asp residue as well as both Ser residues (aa 119, 121, and 123 in the human β3-integrin) are essential [43]. A slightly different motif is present in Smβ-Int1 (DLSYTMIDDLET). While the first Asp and Ser residues (aa 119/121 in human, aa 143/145 in Smβ-Int1) are conserved, a Thr residue replaces the second Ser residue (aa 123 in human, aa 147 in Smβ-Int1) in Smβ-Int1. A mutation of the second Ser residue in human β3-integrin resulted in the loss of its RGD-binding capacity [43]. Thus it seems likely that Smβ-Int1 binds ligands independent of, or different from the RGD motif. The same mutation occurs also in *S. japonicum* (Sjp_0081260), *S. haematobium* (Sha_105750), *C. sinensis* β-integrin 1 (DLSYTMSDDLET), as well as within the β-integrins 1 from *E. multilocularis* and *E. granulosus* (DQSYTMRDDLET). Interestingly, the RGD-binding motif is conserved within the β-integrin of *S. mediterranea* (DLSYSMKDDLKT) indicating that the loss of the RGD-binding capacity and/or the binding of RGD-less ligands emerges as a signature specific for β-integrins of parasitic platyhelminthes. In other parasitic worms, such as the nematodes *Strongyloides ratti* or *Nocardia brasiliensis*, the RGD-binding motif is also well conserved and shows no mutation (DLSYSMKDDKQK in *S. ratti*; DLSYSMKDDKQK in the *N. brasiliensis*). This applies also the closest human ortholog, beta-integrin 4.

In conclusion, β-integrins from parasitic platyhelminthes might bind ligands different from typical RGD-ligands like fibronectin. This view is indirectly supported by the fact that no fibronectin homolog could be identified in schistosomes or *C. sinensis*. The platyhelminth β-integrins are closely related to the human β4 subunit, which is a laminin-binding integrin [6]. Indeed, laminin homologs were identified in *S. mansoni* (Smp_143680, Smp_148790, Smp_163810, Smp_128590) thus representing potential ligands. The human β4-integrin is also known to contact intermediate filaments via fibronectin III-type domains [4], which are present in schistosome proteins (e.g.: Smp_175050, factor for adipocyte differentiation; Smp_126240, titin).

Smβ-Int1 and Smα-Int1 were localized in a variety of tissues of adult schistosomes, like the gonads (ovary, vitellarium, testes), the ootype-surrounding area, the subtegument, and some cells of the parenchyma, whereas Smα-Int2 transcripts were localized in the ootype-surrounding area. Concluding from these results, a Smβ-Int1/Smα-Int1 heterodimer may be present in the gonads, whereas a Smβ-Int1/Smα-Int2 heterodimer might fulfill more specific functions in the ootype-surrounding area. For Smα-Int3 and Smα-Int4, sense and antisense transcripts were detected in the ovary, indicating that the expression of these two α-integrins is presumably organ-specific and regulated by antisense-RNA, a phenomenon for which increasing evidence has been obtained also in schistosomes [50], [61].

Within the ovary and the testes Smβ-Int1/Smα-Int1 co-localized with transcripts of the Src kinase SmTK3, the Src/Abl kinase SmTK6, and the Syk kinase SmTK4 (the latter two are not transcribed in the vitellarium). In cell culture studies, first evidence for β-integrin - Src or Syk kinase interactions were obtained as well as evidence for their involvement in cytoskeletal reorganization events initiated by outside-in signaling [8], [11], [51], [62]. Recently, we identified and confirmed interactions between the Syk kinase SmTK4 and the Src kinase SmTK3 as well as the Src/Abl kinase SmTK6 pointing to a kinase complex acting in the gonads of *S. mansoni* [27], [29]. As a first receptor activating this complex, the atypical RTK SmVKR1 was identified, whose transcripts co-localized in the ovary [27], [31]. Since RTKs and integrins often cluster to integrate signaling pathways [4]–[5], [12], [63], and since it has been described that Syk and Src kinases cooperatively interact with the intracellular parts of β-integrins to adopt catalytical functions [51], Smβ-Int1 may represent an additional upstream interaction candidate of this kinase complex. One early event in integrin signaling is the binding of the tandem SH2-domains of Syk to the intracellular parts of β-integrins [11]. Compared to schistosome Src kinase interactions we found SmTK4-SH2SH2 binding to the C-term of Smβ-Int1 to be strong, which was confirmed by co-immunoprecipitation.

**Figure 7:**
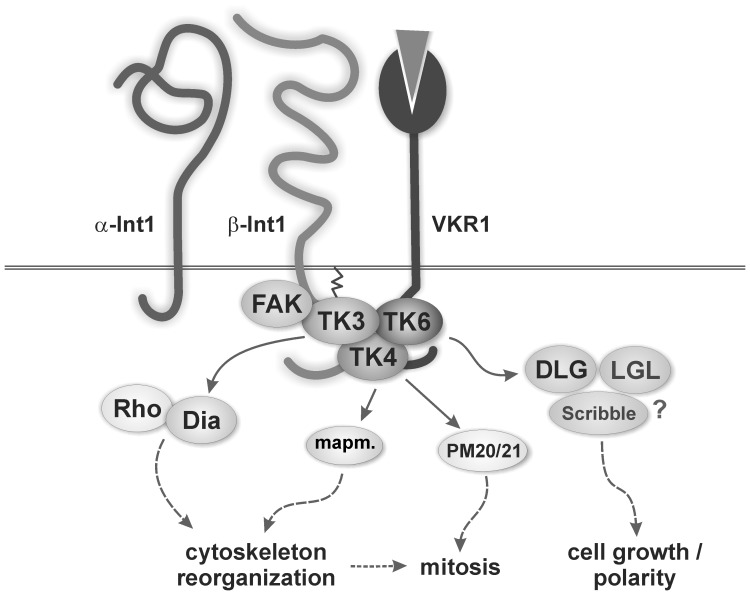
Model for integrin receptor and RTK-induced signaling pathways in *S. mansoni*. The Syk kinase SmTK4, but also the Src kinase SmTK3, and the Src/Abl kinase SmTK6 are able to bind to the intracellular part of Smβ-Int1. Results of previous studies had already indicated that these three kinases interacted with each other and with SmVKR1, and all co-localized in the ovary of females [23], [27], [29]. Furthermore, SmTK3 was found to interact with the diaphanous homolog SmDia [71], which is a binding partner of the Rho-GTPase SmRho1. Both SmDia and SmRho1 were suggested to organize the actin cytoskeleton within the gonads of schistosomes [71]. As downstream partners of SmTK4, MAPK-activating protein (PM20/21) and mapmodulin were found, which may be involved in cytoskeleton reorganization and mitosis [29]. SmDLG as a binding partner of SmTK6 [27] may become activated upon complex formation and may subsequently interact with SmLGL and Scribble to control processes of cell growth and/or cell polarity (Buro et al., unpublished).

Based on our data and todaýs knowledge about RTKs involved in signalling processes controlling mitogenic activity [64], but also cytoskeleton reorganization in cooperation with integrins [65], we postulate the following scenario (Fig. 7). Upon binding of a yet unknown ligand, clustering of the Smβ-Int1 leads to an increase in the local concentration of membrane-associated kinases [27]. In cooperation with VKR1 a Src-Syk kinase complex is formed, in which SmTK4 binds by its tandem SH2-domain to Smβ-Int1 and gets activated by SmTK3 and/or SmTK6. SmTK4 then forwards the signal to downstream targets like a mapmodulin homolog and/or a MAPK-activating protein (PM20/21), which previously had been identified as potential downstream binding partners [29], regulating proliferation and/or differentiation processes in the schistosome gonads SmTK6 itself can also bind to a Discs large homolog from *S. mansoni* (SmDLG), which may subsequently interact with a lethal giant larvae homolog (SmLGL) and Scribble to control processes of cell growth and/or cell polarity ([27]; Buro et al., unpublished).

For functional analyses of the *S. mansoni* integrins we made use of Echistatin, a potent integrin inhibitor [52]–[53]. After treatment of adult worms we observed no physiological or morphological changes, which is explained by the above mentioned mutation in the RGD-binding motif in the β-integrin subunit of *S. mansoni*. RNAi approaches with dsRNAs or individual siRNA molecules to post-transcriptionally silence Smα-Int1 or Smβ-Int1 led only to a minor reduction of their transcript levels (about 20%) without any phenotypic changes in treated worms. Surprisingly, combining all four siRNAs succeeded in silencing Smβ-Int1 activity down to 58%, whereas an analogous approach trying to knock down Smα-Int1 failed. It was shown before that RNAi works in the tegument, the gastrodermis and even the gonads of adult schistosomes [29], [56], [66]. However, not all schistosome genes can be suppressed to the same extent, or cannot be suppressed at all, the latter genes being currently categorized as “non-knockables” [56]. This may also apply for Smα-Int1 and SmTK6 [27], two genes which have eluded silencing approaches in our hands so far. In the case of Smβ-Int1, the combinatorial siRNA approach helped to overcome the limitations using dsRNA or individual siRNAs. Although this is highly speculative at present, the differences observed could be explained assuming that there are subsets of the RNAi machinery responding differently to siRNAs. If so, some siRNAs would be recognized exclusively or more efficiently by one “specialized” subset but not by another, and vice versa. Saturating different subsets at the same time may eventually exceed a hypothetical threshold level needed to achieve efficient knock-down. Although further research is needed to uncover the observed effects, the combinatory approach nevertheless opens a novel perspective for silencing approaches of genes that have refused responding to existing knock-down protocols so far. Within the ovaries of most of the treated females a slightly increased number of big mature oocytes were found to be distributed also in the anterior part of the ovary, which in wildtype worms contains only small immature oocytes [23], [48]. Reduced Smβ-Int1 function may have resulted in a defect in oocyte transportation within the ovary. This phenotype corresponded to that observed after inhibiting SmTK4 function by the inhibitor Piceatannol or SmTK4-specific dsRNAs in the ovary of females [29]. In connection with the co-immunoprecipitation data confirming Smβ-Int1-SmTK4 interaction and their co-localization, these data additionally support the assumption that both molecules act in the same pathway (Fig. 7). Additionally, Smβ-Int1 knock-down led to some multinucleated oocytes, which we never have seen so far in other knock-down approaches or any controls. Recently, integrins were identified as regulators of mitotic events and described to regulate spindle positioning and orientation. Importantly, it was demonstrated that perturbing integrin function resulted in the generation of multipolar spindles leading to aneuploidy [67]. Furthermore, chimeric mice carrying α/β-integrin deficiency exhibited bi- and multi-nucleated cells pointing to a function of β-integrins in cytokinesis [68]. Thus, the Smβ-Int1 knock-down phenotype observed indicates a similar function in schistosome oocytes. Not all oocytes revealed this phenotype, which is explained by the partial Smβ-Int1 knock-down. Besides integrins, also the Rho family of small GTPases is known to have a central function in cytoskeletal organization in mammalian cells [69]. Rho GTPases were described to act together with Src, Syk, and other proteins in an α/β-integrin-stimulated signaling complexes organizing the cytoskeleton [70]. We have recently shown that schistosome diaphanous (SmDia) is a downstream interaction partner of SmTK3, but also interacts with the schistosome Rho GTPase SmRho1 [71]. Dia proteins on their parts are known to be also involved in cytoskeleton organization events [72], [73]. An RNAi approach to knock down SmDia with specific dsRNAs resulted in transcript reduction down to 43% (Fig. 6B), which also led to multinucleated oocytes within the ovary (Fig. 6, E, F), which perfectly corresponded to the Smβ-Int1 knock-down phenotype. Functional links between integrin and Rho GTPase signaling were reported before, among others for gonad differentiation processes in *C. elegans* [74]–[75]. Thus, we conclude that integrin and small GTPase (Rho) signaling in the ovary of the schistosome female might cooperate in some way to control oocyte cytoskeleton reorganization and/or mitosis/meiosis.

This study provides a first comprehensive analysis of platyhelminth integrins revealing its exceptional nature, especially of the parasite orthologs that significantly differ from their counterparts in free-living flatworms. Our molecular analysis of integrins from a model trematode provided first insights into the functions of these receptors in reproductive processes of schistosomes. Together with the supportive evidence of cooperation between integrin and RTK signaling [4], [12], our findings shed new light on the complexity of the cellular processes involved in the reproductive biology of trematodes, which may be representative for other platyhelminthes as well.

## Materials and Methods

### Ethics Statement

All experiments involving hamsters as final hosts to maintain the schistosome life cycle have been performed in accordance with the European Convention for the Protection of Vertebrate Animals used for Experimental and other Scientific Purposes (ETS No 123; revised Appendix A), and have been approved by the Regional Council (Regierungspraesidium) Giessen (V54-19c 20/15 (1) GI 20(8 Nr. A 6/2011).

### Parasite Stock

A Liberian isolate of *S. mansoni* was maintained in *Biomphalaria glabrata* as intermediate host and in Syrian hamster (*Mesocricetus auratus*) as definitive host [76]. Adult worms were obtained by hepatoportal perfusion at 42–49 days post-infection.

### *In silico* Identification of Integrin Receptors from *S. mansoni* and other Platyhelminthes

For the identification of integrin receptors from schistosomes BLAST analyses of the *S. mansoni* genome data set were performed (www.sanger.ac.uk) [37]. To this end heterologous α- and β-integrin receptor sequences from e.g. *Mus musculus* were used as template for a *S. mansoni* omniblast (http://www.sanger.ac.uk/cgi-bin/blast/submitblast/s_mansoni/omni). Sequences with homology to integrins were further analyzed using the following public domain tools: NCBI-BLAST (http://blast.ncbi.nlm.nih.gov/Blast.cgi), SMART (http://smart.embl-heidelberg.de), Signal-3L, the Wellcome Trust Sanger Institute *S. mansoni* OmniBlast server (http://www.sanger.ac.uk/cgi-bin/blast/submitblast/s_mansoni/omni), SchistoDB 3.0 (http://beta.schistodb.net/schisto.beta), GeneDB (http://www.genedb.org), and SmedGD v1.3.14 (http://smedgd.neuro. utah.edu).

### Cloning of Integrin Receptors

To amplify the coding sequences of the *in silico*-identified integrins, total RNA from *S. mansoni* was extracted using TriFast (PeqLab) following the manufacturer’s instructions. cDNA was synthesized using the QuantiTect reverse transcription system (Qiagen) with 1 µg total RNA as template, a mixture of oligo-d(T) primer and random hexamer primers, and Quantiscript reverse transcriptase (Qiagen). Subsequent PCRs were performed with 1/4 of the cDNA as template, and FIREPol *Taq* polymerase (Solis BioDyne).

The coding sequence for the first α-integrin Smα-Int1 was amplified in two overlapping fragments (2051 bp, 1907 bp) with the primer combinations α-Int1-5′ (5′-ATGGATCTTACATTTCCATTTTG-3′)+α-Int1-int-3′ (3′-ATCATTTGAACTTGTAAATCAGGT-3′), and α-Int1-int-5′ (5′-AACACAAATCAATATCATCATGG-3′)+α-Int1-3′ (5′-TTAATTTTTTCTCGGTTCAATAAAT-3′). For the amplification of the second α-integrin Smα-Int2, also in two overlapping fragments (2319 bp, 2229 bp), the primers α-Int2-fwd1 (5′-ATGTTCATTTGGAATATTTATTTTATAC-3‘)+α-Int2-rev1 (5′-TTCAGACCAATCAATTAAAGG-3‘) and α-Int2-fwd2 (5′-GAATGTAATTTTATGCAAAATCTTG-3′)+α-Int2-rev2 (5′-TCACAATTCAAAATTGTTGTTAAG-3‘) were used. The coding sequence for the third α-integrin Smα-Int3 was amplified in three overlapping fragments (1379 bp, 1451 bp, 1061 bp) using the primer pairs α-Int3-fl-fwd1 (5′-ATGGGATATACTCACTTTTCAGG-3′)+α-Int3-fl-rev1 (5′-TCCATTAATGTTAAATATGCGC-3′), α-Int3-fl-fwd2 (5′-GCTCTGTTGCAGTGTTACG-3′)+α-Int3-fl-rev2 (5′-ATGTGGTTCATTGATAAGCC-3′), and α-Int3-fl-fwd3 (5′-GTCTGATCATCGATTGGC-3′)+α-Int3-fl-rev3 (5′-CTATTTATGTGAAATAGGAAAGTCAGAT-3′). The fourth α-integrin Smα-Int4 was amplified in four overlapping fragments (1333 bp, 1279 bp, 1252 bp, 695 bp) using the following primer combinations: α-Int4-fl-fwd1 (5′-ATGTGGATTACTACTAAATTCTATCAAAC-3′)+α-Int4-fl-rev1 (5′-TTTTGATATCTGTATTACCGGC-3′), α-Int4-fl-fwd2 (5′-CATCAAAAGTGTTATCATGGG-3′)+α-Int4-fl-rev2 (5′-TTGTGCTTTTGTTGGTCC-3′), α-Int4-fl-fwd3 (5′-GGTAATCGAAATAGTTCTAGACCAC-3′)+α-Int4-fl-rev3 (5′-CAAACAGAGTACGTGCCC-3′), and α-Int4-fl-fwd4 (5′-CTGTTTTAATTAAAGTTACTGGATGG-3′)+α-Int4-fl-rev4 (5′-TTATTGTGATGAAGTAGAATCTGTAATTA-3′). The primers β-Int1-5′ (5′-ATGGGGATACGCCCTGC-3′)+β-Int1-int-3′ (5′-TGTCTGAACACCTAGTACAGCTT-3′), and β-Int1-int-5′ (5′-GCTAATGGCTACTTATGATGCAG-3′)+β-Int-3′ (5′-TCAATATCCGTTTTCTTCAAAAG-3′) were used for the amplification of the coding sequence of the β-integrin Smβ-Int1 in two overlapping fragments (1398 bp, 1423 bp). Amplification products of the expected sizes were cloned into pDrive (Qiagen) and sequenced commercially (LGC genomics, Berlin).

### Localization Studies

*In situ* hybridizations were performed as described in detail elsewhere [26], [71]. In short, adult worm pairs were fixed in Bouin’s solution (picric acid/acetic acid/formaldehyde; 15/1/5) before embedding in paraplast (Histowax, Reichert-Jung). Sections of 5 µm were generated and incubated in xylol to remove the paraplast. Following re-hydration, proteins were removed by proteinase K treatment (final concentration 1 µg/ml), and the sections were dehydrated. For hybridization, *in vitro* transcripts were labeled with digoxigenin following the manufacturerś instructions (Roche). Labeled sense and antisense transcripts of the integrin (positions 3073–3528 (Smα-Int1), 3696–4167 (Smα-Int2), 1798–2141 (Smα-Int3), 1691–2032 (Smα-Int4), and 1875–2177 (Smβ-Int1)) were size-controlled by gel electrophoresis. To prove their quality, transcript blots were made confirming digoxigenin incorporation by alkaline phosphatase-conjugated anti-digoxigenin antibodies (Roche), naphthol-AS-phosphate, and Fast Red TR (Sigma). All *in situ* hybridizations were performed for 16 h at 42°C. Sections were stringently washed up to 0.5 x SSC, and detection was achieved as described for transcript blots.

### Direct YTH Interaction Studies

For interaction studies in the yeast two-hybrid system (YTH), yeast cells of the strain AH109 (Mat a; reporter genes ADE2, HIS3, and LacZ) were co-transformed with appropriate bait and prey plasmids by the lithium acetate method according to the user manual (Yeast protocols handbook, Clontech). As bait vector, the plasmid pBridge (tryptophan nutritional marker TRP1, Clontech) was used, which allows the cloning of protein-coding gene sequences as fusion constructs with the GAL4 DNA binding domain (GAL4-BD). As prey vector, the plasmid pACT2 (leucine nutritional marker LEU2, Clontech) was used, which allows the expression of proteins as fusion with the GAL4 activation domain (GAL4-AD).

After co-transformation, yeast cells were selected for diploid cells containing interacting proteins on synthetic dropout medium lacking the amino acids tryptophan, leucine, and histidine (Trp^−^/Leu^−^/His^−^). Qualitative detection of LacZ expression was performed using β-galactosidase (β-Gal) colony lift filter assays and for quantification of relative interaction strengths, β-Gal liquid assays with ONPG (o-nitrophenol-galactopyranoside, Sigma) as substrate were performed, both according to the Yeast protocols handbook from Clontech.

### Cloning of Bait and Prey Vectors

Interaction studies with the β-integrin were performed with a prey vector containing the intracellular, C-terminal part of Smβ-Int1 cloned into pACT2 in frame with the GAL4-AD. The encoding sequence was amplified by PCR using the primer pair β-Int1-C-term-5′ (5′-GGATCCGAAAACTAGTCATTACAATTGATGA-3′; containing a *Bam*HI restriction site) and β-Int1-C-term-3′ (5′-CTCGAGTTATCAATATCCGTTTTCTTCAAAAGTTG-3′; containing a *Xho*I restriction site and a stop codon), and an appropriate cDNA clone as template. Amplification products of the expected size (167 bp) were cloned *via Bam*HI/*Xho*I into pACT2. The resulting construct Smβ-Int1-C-term pACT2 was sequenced confirming the correct open reading frame of the GAL4-AD/Smβ-Int1-C-terminus fusion.

As bait vectors, the relevant protein interaction domains of the CTKs SmTK4, SmTK6, and SmTK3 were cloned into pBridge in frame with the Gal4-BD. The cloning of the first bait construct SmTK4-SH2SH2 pBridge was described elsewhere [29]. For the amplification of the combined SH4SH3 domains or single SH3 domains of SmTK6 and SmTK3 by PCR, appropriate template cDNA clones and the following primer combinations were used: TK6-SH4SH3-5′ (5′-GGATCCGTATGGGAATTTGTTTGTGTCTTC-3′; containing a *Bam*HI restriction site)+TK6-SH3-3′ (5′-CTGCAGTCAAGTTGGAAGTCCATCATTCAG-3′; containing a *Pst*I restriction site and a stop codon), TK6-SH3-5′ (5′-GGATCCGTTTGGTACAGGTTCGCGCTC-3′; containing a *Bam*HI restriction site)+TK6-SH3-3′, TK3-SH4SH3-5′ (5′-GGATCCGTATGGGAAATTCTAATTCGTCTAA-3′; containing a *Bam*HI restriction site)+TK3-SH3-3′ (5′-CTGCAGTCATTCCAAACTGGTAACAGCTG-3′; containing a *Pst*I restriction site and a stop codon), and TK3-SH3-5′ (5′-GGATCCGTACAGAAGGGCAGTTTGTTGC-3′; containing a *Bam*HI restriction site)+TK3-SH3-3′. Amplification products of the expected sizes (TK6-SH4SH3 539 bp; TK3-SH4SH3 658 bp; TK6−/TK3-SH3 206 bp) were cloned *via Bam*HI/*Pst*I into the vector pBridge. After cloning, the resulting constructs SmTK6-SH4SH3 pBridge, SmTK6-SH3 pBridge, SmTK3-SH4SH3 pBridge, and SmTK3-SH3 pBridge were sequenced confirming the correct open reading frame of the GAL4-BD fusions.

### Co-immunoprecipitation of Smβ-Int1-C-term with SmTK4 after Expression in *Xenopus* Oocytes

A part of Smβ-Int1 containing the C-terminus but no transmembrane domain and an HA-tag at its N-terminus (Smβ-Int1-C-term; 750 bp, 250AA) was subcloned into pcDNA 3.1. Capped messenger RNA (cRNA) encoding Smβ-Int1-C-term was synthesized *in vitro* using the T7 mMessage machine Kit (Ambion, USA) as previously described [77]. cRNA encoding full-length V5-tagged SmTK4 [29] was prepared using the same procedure. Interaction studies between Smβ-Int1-C-term and SmTK4 were performed by co-injection of both cRNAs in *Xenopus* oocytes. Expressed proteins were detected by immunoprecipitation of lysates as previously described [77]. In short, after 24 h of expression, 30 oocytes were lysed in 300 µl of buffer (50 mM HEPES, pH 7.4, 500 mM NaCl, 5 mM MgCl_2,_ 1 µg/ml bovine serum albumin, 10 µg/ml leupeptin, 10 µg/ml aprotinin, 10 µg/ml soybean trypsin inhibitor, 10 µg/ml benzamidine, 1 mM PMSF, 1 mM sodium vanadate) and submitted to centrifugation at 4°C for 15 min at 10,000 g. The resulting supernatants were incubated with anti-V5 (1∶100; Invitrogen) or anti-HA (1∶100; Invitrogen) for 2 h at 4°C, then added to protein A-Sepharose beads (5 mg, Amersham Biosciences) for 1 h at 4°C. Beads were washed three times and immune complexes were eluted in Laemmli buffer and analyzed in 10% polyacrylamide gels by SDS-PAGE. Western blot analyses were performed with anti-V5 or anti HA (1∶50 000) antibodies and detection performed using the advanced ECL detection system (Amersham Biosciences).

### *In vitro* Culture of Adult Schistosomes, Inhibitor Treatment and Morphological Analysis

After perfusion, adult schistosomes were washed three times with M199 medium before being cultured *in vitro* in M199 (Gibco; including glucose, sodium bicarbonate, 4-(2-hydroxyethyl)-1-piperazineethane sulfonic acid) supplemented with an antibiotic/antimycotic mixture (1.25%, Sigma) and FCS (10%, Gibco) at 37°C and 5% CO_2_ [23].

For integrin inhibition experiments the disintegrin Echistatin was used [53]–[55]. For each inhibitor treatment experiment 10 couples were maintained in 60 mm diameter culture dishes in 5 mL culture medium, supplemented with 100 nM or 500 nM Echistatin (Sigma, dissolved in water, 0.1 mg/ml). Medium and inhibitor were refreshed every 24 hours during the *in vitro* treatment periods (3–5 days). For morphological analysis, adult worms were fixed for at least 24 hours in AFA (alcohol 95%, formalin 3%, and glacial acetic acid 2%), stained for 30 minutes with 2.5% hydrochloric carmine (Certistain®, Merck), and destained in acidic 70% ethanol. After dehydration for 5 minutes in 70%, 90%, and 100% ethanol, worms were preserved as whole-mounts in Canada balsam (Merck) on glass slides [29], [48]. CLSM images were made on a Leica TSC SP2 microscope using a 488 nm He/Ne laser and a 470 nm long-pass filter in reflection mode [23], [29].

### RNAi Experiments

As basis for double-stranded RNA (dsRNA) synthesis, a 901 bp fragment of the Smβ-Int1-coding DNA was amplified by PCR using the gene-specific primers β-Int1-dsRNA-5′ (5′-TCGTTGTGCTCCATCTTCAG-3′)+β-Int1-dsRNA-3′ (5′-TGCACTTGGCAAGAATTCAG-3′). For Smα-Int1, a 919 bp fragment was amplified using the gene-specific primers α-Int1-dsRNA-5′ (5′-CGACAGCATTTTCAGTCAAAC-3′)+α-Int1-dsRNA-3′ (5′-GTGTTGGCAGTTCCCAATTT-3′). The primers DiaRNAi1-5' (5′-CCACCGTCATCGGCAAATATG-3′)+DiaRNAi1-3' (5′-GCGCAACTTCAGGACAATCC-3′), and DiaRNAi2-5' (5′-TGGGGAGCAAATCAGAGAAGC-3′)+DiaRNAi2-3' (5′-CTCAAGCTCATCCTCATGAAG-3′) were used to amplify 670 bp and 631 bp fragments of the *S. mansoni* Diaphanous homolog SmDia [71] as template for dsRNA synthesis. For all PCR reactions appropriate cDNA clones were used as DNA templates. Amplification products of the expected sizes were cloned into pDrive (Qiagen). The resulting constructs, containing T7 and SP6 RNA polymerase promoters flanking the integrin or SmDia sequences, were used to generate single-stranded RNAs by *in vitro* transcriptions with T7 and SP6 RNA polymerases (MEGAscript RNA transcription kit, Ambion). The single-stranded RNAs were purified by LiCl-precipitation, resuspended in dH_2_O, and quantified by spectrophotometry. Equal amounts of the single-stranded RNAs were mixed in annealing buffer (500 mM potassium acetate, 150 mM HEPES-KOH, pH 7.4, 19 mM magnesium acetate, sterile dH_2_O) and incubated at 68°C for 15 min. Annealing and integrity of the dsRNAs were confirmed by agarose gel electrophoresis.

In addition to dsRNA, four specific small inhibitory RNAs (siRNAs) were designed for Smβ-Int1 and Smα-Int1 with the help of the on-line IDT RNAi Design Tool (https://eu.idtdna.com/Scitools/Applications/RNAi/RNAi.aspx) and synthesized commercially (Riboxx GmbH) with the following sense-strand sequences: beta-integrin1-siRNA1 5'-AUAAAGCAGAUUCACCACCCCC-3', beta-integrin1-siRNA2 5'-AUAAGACUGAAGAUGGAGCCCCC-3', beta-integrin1-siRNA3 5'-UAAUUGACAUCUUGCAGAGCCCCC-3', beta-integrin1-siRNA4 5'-AGUAAUGCGUCCAUACCACCCCC-3', alpha-Integrin1-siRNA1 5'-UAAAUCGUUUGUUUCCGUGCCCCC-3', alpha-Integrin1-siRNA2 5'-AUGUUUAUGUUGUUCACCCCC-3', alpha-Integrin1-siRNA3 5'-UAAUCUGGUAUUUCUGGUCCCCC-3', and alpha-Integrin1-siRNA4 5'-AUUGAUUUAUCUGUUGGACCCCC-3'. The dsRNAs or siRNAs were delivered to adult worms by electroporation according to published protocols [29], [56], [66]. Briefly, electroporations were performed in 4 mm cuvettes with 10 couples each in 50–100 µl electroporation buffer (Ambion) containing 25 µg dsRNA (Smβ-Int1, Smα-Int1), 40 µg dsRNA (20 µg dsRNA1+20 µg dsRNA2 of SmDia) or 2.5 µg siRNA (Smβ-Int1, Smα-Int1). A square-wave protocol was applied with a single 20 ms impulse at 125 V and at room temperature (Gene Pulser XCell™, Biorad). After electroporation, the worms were transferred to complete M199 medium and incubated for 5 days; 48 hours after electroporation the medium was refreshed. In case of the SmDia RNAi approach, the medium was changes at day 2 and 5 after electroporation and dsRNAs were added to the medium for an additional soaking effect.

For transcript detection the following primer pairs were used for RT-PCRs: α-Int1-RTPCR-5′ (5′-CGATATCTCTGAAGGTCCAACA-3′)+α-Int1-RTPCR-3′ (5′-TCGAAATGCTCCAGCTGTATT-3′), β-Int1-RTPCR-5′ (5′-CGATATCTCTGAAGGTC-CAACA-3′)+β-Int1-RTPCR-3′ (5′-TCGAAATGCTCCAGCTGTATT-3′), and SmDia-5.1 (5′-ATTATGGGGAGCAAATCAGA-3′)+SmDia-3.1 (5′-ATCCCCATACTCAAGCTCA-3′). For normalization, the transcription of the housekeeping gene SmPDI [78] was monitored using the same cDNAs as template and the following primer combination: SmPDI-5′ (5′-GGGATTTATCAAGGATACGGACTC-3′) and SmPDI-3′ (5′-CACCAAGGAGCATACAG-TTTGAC-3′). All PCRs were performed in a final volume of 25 µl. PCR products were separated on 1.5% agarose gels stained with ethidium bromide. The relative intensities of the amplification products were determined densitometrically using the program ImageJ (version 1.4.1; http://rsbweb.nih.gov/ij/index.html). For relative quantification of the integrin RT-PCR products, the SmPDI amplicons were used as endogenous standard.

## Supporting Information

Table S1 Protein domains of the α/β- integrin receptors from different parasitic plathyhelminths and human. Protein domains of the α- (**A**) and β- (**B**) integrin receptors from *S. mansoni* (Sm), *S. haematobium* (Sha), *S. japonicum* (Sjp), *C. sinensis* (Cs), *E. multilocularis* (Em), *S. mediterranea* (Smed), and *H. sapiens* (Hs) as predicted by SMART analyses and signal peptide prediction. ^#^: NCBI accession number; ^*^: GeneDB number, °: SmedDB number, ^+^: SwissProt number, n.p.: not predicted.
(DOCX)

## References

[1] Fu G, Wang W, Luo BH (2012) Overview: structural biology of integrins. Methods Mol Biol 757: 81–99. 21909908 10.1007/978-1-61779-166-6_7

[2] Anthis NJ, Campbell ID (2011) The tail of integrin activation. Trends Biochem Sci 36: 191–198. 21216149 10.1016/j.tibs.2010.11.002 PMC3078336

[3] Kim C, Ye F, Ginsberg MH (2011) Regulation of integrin activation. Annu Rev Cell Dev Biol 27: 321–345. 21663444 10.1146/annurev-cellbio-100109-104104

[4] Campbell ID, Humphries MJ (2011) Integrin structure, activation, and interactions. Cold Spring Harb Perspect Biol 3: pii, a004994. 10.1101/cshperspect.a004994 PMC3039929 21421922

[5] Guo W, Giancotti FG (2004) Integrin signalling during tumour progression. Nat Rev Mol Cell Biol 5: 816–826. 15459662 10.1038/nrm1490

[6] Hynes RO (2002) Integrins: bidirectional, allosteric signaling machines. Cell 110: 673–687. 12297042 10.1016/s0092-8674(02)00971-6

[7] Jakus Z, Fodor S, Abram CL, Lowell CA, Mocsai A (2007) Immunoreceptor-like signaling by beta 2 and beta 3 integrins. Trends Cell Biol 17: 493–501. 17913496 10.1016/j.tcb.2007.09.001

[8] Obergfell A, Eto K, Mocsai A, Buensuceso C, Moores SL, et al (2002) Coordinate interactions of Csk, Src, and Syk kinases with [alpha]IIb[beta]3 initiate integrin signaling to the cytoskeleton. J Cell Biol 157: 265–275. 11940607 10.1083/jcb.200112113 PMC2199242

[9] Soung YH, Clifford JL, Chung J (2010) Crosstalk between integrin and receptor tyrosine kinase signaling in breast carcinoma progression. BMB Rep 43: 311–318. 20510013 10.5483/bmbrep.2010.43.5.311

[10] Mitra SK, Schlaepfer DD (2006) Integrin-regulated FAK-Src signaling in normal and cancer cells. Curr Opin Cell Biol 18: 516–523. 16919435 10.1016/j.ceb.2006.08.011

[11] Woodside DG, Obergfell A, Talapatra A, Calderwood DA, Shattil SJ, et al (2002) The N-terminal SH2 domains of Syk and ZAP-70 mediate phosphotyrosine-independent binding to integrin beta cytoplasmic domains. J Biol Chem 277: 39401–39408. 12171941 10.1074/jbc.M207657200

[12] Ivaska J, Heino J (2011) Cooperation between integrins and growth factor receptors in signaling and endocytosis. Annu Rev Cell Dev Biol 27: 291–320. 21663443 10.1146/annurev-cellbio-092910-154017

[13] Chitsulo L, LoVerde P, Engels D (2004) Schistosomiasis. Nat Rev Microbiol 2: 12–13. 15035004 10.1038/nrmicro801

[14] Engels D, Chitsulo L, Montresor A, Savioli L (2002) The global epidemiological situation of schistosomiasis and new approaches to control and research. Acta Trop 82: 139–146. 12020886 10.1016/s0001-706x(02)00045-1 PMC5633073

[15] Quack T, Beckmann S, Grevelding CG (2006) Schistosomiasis and the molecular biology of the male-female interaction of *S. mansoni* . Berl Munch Tierarztl Wochenschr 119: 365–372. 17007463

[16] Ross AG, Bartley PB, Sleigh AC, Olds GR, Li Y, et al (2002) Schistosomiasis. N Engl J Med 346: 1212–1220. 11961151 10.1056/NEJMra012396

[17] Erasmus DA (1973) A comparative study of the reproductive system of mature, immature and “unisexual” female *Schistosoma mansoni* . Parasitology 67: 165–183. 4795964 10.1017/s0031182000046394

[18] Grevelding CG (2004) *Schistosoma* . Curr Biol 14: R545. 15268869 10.1016/j.cub.2004.07.006

[19] Knobloch J, Kunz W, Grevelding CG (2002) Quantification of DNA synthesis in multicellular organisms by a combined DAPI and BrdU technique. Dev Growth Differ 44: 559–563. 12492514 10.1046/j.1440-169x.2002.00667.x

[20] Kunz W (2001) Schistosome male-female interaction: induction of germ-cell differentiation. Trends Parasitol 17: 227–231. 11323306 10.1016/s1471-4922(01)01893-1

[21] LoVerde PT, Niles EG, Osman A, Wu W (2004) *Schistosoma mansoni* male-female interactions. Can J Zool 82: 357–374.

[22] Popiel I (1986) Male-stimulated female maturation in *Schistosoma*: a review. J Chem Ecol 12: 1745–1754. 24305892 10.1007/BF01022380

[23] Beckmann S, Quack T, Burmeister C, Buro C, Long T, et al (2010) *Schistosoma mansoni*: signal transduction processes during the development of the reproductive organs. Parasitology 139: 651–668. 10.1017/S0031182010000053 20163751

[24] Knobloch J, Beckmann S, Burmeister C, Quack T, Grevelding CG (2007) Tyrosine kinase and cooperative TGFbeta signaling in the reproductive organs of *Schistosoma mansoni* . Exp Parasitol 117: 318–336. 17553494 10.1016/j.exppara.2007.04.006

[25] LoVerde PT, Osman A, Hinck A (2007) *Schistosoma mansoni*: TGF-beta signaling pathways. Exp Parasitol 117: 304–317. 17643432 10.1016/j.exppara.2007.06.002 PMC2149906

[26] Kapp K, Knobloch J, Schussler P, Sroka S, Lammers R, et al (2004) The *Schistosoma mansoni* Src kinase TK3 is expressed in the gonads and likely involved in cytoskeletal organization. Mol Biochem Parasitol 138: 171–182. 15555729 10.1016/j.molbiopara.2004.07.010

[27] Beckmann S, Hahnel S, Cailliau K, Vanderstraete M, Browaeys E, et al (2011) Characterization of the SRC/ABl hybrid-kinase SMTK6 of *Schistosoma mansoni* . J Biol Chem 286: 42325–42336. 22013071 10.1074/jbc.M110.210336 PMC3234968

[28] Beckmann S, Grevelding CG (2010) Imatinib has a fatal impact on morphology, pairing stability and survival of adult *Schistosoma mansoni in vitro* . Int J Parasitol 40: 521–526. 20149792 10.1016/j.ijpara.2010.01.007

[29] Beckmann S, Buro C, Dissous C, Hirzmann J, Grevelding CG (2010) The Syk Kinase SmTK4 of *Schistosoma mansoni* Is Involved in the Regulation of Spermatogenesis and Oogenesis. PLoS Pathog 6: e1000769. 20169182 10.1371/journal.ppat.1000769 PMC2820527

[30] Knobloch J, Winnen R, Quack M, Kunz W, Grevelding CG (2002) A novel Syk-family tyrosine kinase from *Schistosoma mansoni* which is preferentially transcribed in reproductive organs. Gene 294: 87–97. 12234670 10.1016/s0378-1119(02)00760-6

[31] Vicogne J, Pin JP, Lardans V, Capron M, Noel C, et al (2003) An unusual receptor tyrosine kinase of *Schistosoma mansoni* contains a Venus Flytrap module. Mol Biochem Parasitol 126: 51–62. 12554084 10.1016/s0166-6851(02)00249-9

[32] Arias-Salgado EG, Lizano S, Sarkar S, Brugge JS, Ginsberg MH, et al (2003) Src kinase activation by direct interaction with the integrin beta cytoplasmic domain. Proc Natl Acad Sci USA 100: 13298–13302. 14593208 10.1073/pnas.2336149100 PMC263791

[33] Kato A, Oshimi K (2009) Ancient ubiquitous protein 1 and Syk link cytoplasmic tails of the integrin alpha(IIb)beta(3). Platelets 20: 105–110. 19235052 10.1080/09537100802641507

[34] Lowell CA (2011) Src-family and Syk kinases in activating and inhibitory pathways in innate immune cells: signaling cross talk. Cold Spring Harb Perspect Biol 3(3): a002352. 21068150 10.1101/cshperspect.a002352 PMC3039931

[35] Piccardoni P, Manarini S, Federico L, Bagoly Z, Pecce R, et al (2004) SRC-dependent outside-in signalling is a key step in the process of autoregulation of beta2 integrins in polymorphonuclear cells. Biochem J 380: 57–65. 14969582 10.1042/BJ20040151 PMC1224154

[36] Totani L, Piccoli A, Manarini S, Federico L, Pecce R, et al (2006) Src-family kinases mediate an outside-in signal necessary for beta2 integrins to achieve full activation and sustain firm adhesion of polymorphonuclear leucocytes tethered on E-selectin. Biochem J 396: 89–98. 16433632 10.1042/BJ20051924 PMC1449987

[37] Berriman M, Haas BJ, LoVerde PT, Wilson RA, Dillon GP, et al (2009) The genome of the blood fluke *Schistosoma mansoni* . Nature 460: 352–358. 19606141 10.1038/nature08160 PMC2756445

[38] Wang X, Chen W, Huang Y, Sun J, Men J, et al (2011) The draft genome of the carcinogenic human liver fluke *Clonorchis sinensis* . Genome Biol 12: R107. 22023798 10.1186/gb-2011-12-10-r107 PMC3333777

[39] Bork P, Doerks T, Springer TA, Snel B (1999) Domains in plexins: links to integrins and transcription factors. Trends Biochem Sci 24: 261–263. 10390613 10.1016/s0968-0004(99)01416-4

[40] Takagi J, Springer TA (2002) Integrin activation and structural rearrangement. Immunol Rev 186: 141–163. 12234369 10.1034/j.1600-065x.2002.18613.x

[41] Calvete JJ, Henschen A, Gonzalez-Rodriguez J (1991) Assignment of disulphide bonds in human platelet GPIIIa. A disulphide pattern for the beta-subunits of the integrin family. Biochem J 274: 63–71. 2001252 10.1042/bj2740063 PMC1150190

[42] Zang Q, Springer TA (2001) Amino acid residues in the PSI domain and cysteine-rich repeats of the integrin beta2 subunit that restrain activation of the integrin alpha(X)beta(2). J Biol Chem 276: 6922–6929. 11096074 10.1074/jbc.M005868200

[43] Bajt ML, Loftus JC (1994) Mutation of a ligand binding domain of beta 3 integrin. Integral role of oxygenated residues in alpha IIb beta 3 (GPIIb-IIIa) receptor function. J Biol Chem 269: 20913–20919. 7520434

[44] Young ND, Jex AR, Li B, Liu S, Yang L, et al (2012) Whole-genome sequence of *Schistosoma haematobium* . Nat Genet 44: 221–225. 22246508 10.1038/ng.1065

[45] *Schistosoma japonicum* Genome Sequencing, Functional Analysis Consortium (2009) The *Schistosoma japonicum* genome reveals features of host-parasite interplay. Nature 460: 345–351. 19606140 10.1038/nature08140 PMC3747554

[46] Robb SM, Ross E, Sanchez AA (2008) SmedGD: the *Schmidtea mediterranea* genome database. Nucleic Acids Res 36: D599–D606. 17881371 10.1093/nar/gkm684 PMC2238899

[47] Hughes AL (2001) Evolution of the integrin alpha and beta protein families. J Mol Evol 52: 63–72. 11139295 10.1007/s002390010134

[48] Neves RH, de Lamare BC, Machado-Silva JR, Carvalho JJ, Branquinho TB, et al (2005) A new description of the reproductive system of *Schistosoma mansoni* (Trematoda: Schistosomatidae) analyzed by confocal laser scanning microscopy. Parasitol Res 95: 43–49. 15565465 10.1007/s00436-004-1241-2

[49] Leutner S, Beckmann S, Grevelding CG (2011) Characterization of the cGMP-dependent protein kinase SmcGK1 of *Schistosoma mansoni* . An Acad Bras Cienc 83: 637–648. 21670884 10.1590/s0001-37652011000200023

[50] Verjovski-Almeida S, Venancio TM, Oliveira KC, Almeida GT, DeMarco R (2007) Use of a 44k oligoarray to explore the transcriptome of *Schistosoma mansoni* adult worms. Exp Parasitol 117: 236–245. 17517391 10.1016/j.exppara.2007.04.005

[51] Zou W, Kitaura H, Reeve J, Long F, Tybulewicz VL, et al (2007) Syk, c-Src, the alphavbeta3 integrin, and ITAM immunoreceptors, in concert, regulate osteoclastic bone resorption. J Cell Biol 176: 877–888. 17353363 10.1083/jcb.200611083 PMC2064061

[52] Gould RJ, Polokoff MA, Friedman PA, Huang TF, Holt JC, et al (1990) Disintegrins: a family of integrin inhibitory proteins from viper venoms. Proc Soc Exp Biol Med 195: 168–171. 2236100 10.3181/00379727-195-43129b

[53] Kumar CC, Nie H, Rogers CP, Malkowski M, Maxwell E, et al (1997) Biochemical characterization of the binding of echistatin to integrin alphavbeta3 receptor. J Pharmacol Exp Ther 283: 843–853. 9353406

[54] Musial J, Niewiarowski S, Rucinski B, Stewart GJ, Cook JJ, et al (1990) Inhibition of platelet adhesion to surfaces of extracorporeal circuits by disintegrins. RGD-containing peptides from viper venoms. Circulation 82: 261–273. 2364514 10.1161/01.cir.82.1.261

[55] Sato M, Sardana MK, Grasser WA, Garsky VM, Murray JM, et al (1990) Echistatin is a potent inhibitor of bone resorption in culture. J Cell Biol 111: 1713–1723. 2211834 10.1083/jcb.111.4.1713 PMC2116239

[56] Krautz-Peterson G, Bhardwaj R, Faghiri Z, Tararam CA, Skelly PJ (2010) RNA interference in schistosomes: machinery and methodology. Parasitology 137: 485–495. 19765345 10.1017/S0031182009991168 PMC2830314

[57] Ewan R, Huxley-Jones J, Mould AP, Humphries MJ, Robertson DL, et al (2005) The integrins of the urochordate *Ciona intestinalis* provide novel insights into the molecular evolution of the vertebrate integrin family. BMC Evol Biol 5: 31. 15892888 10.1186/1471-2148-5-31 PMC1145181

[58] Brower DL, Brower SM, Hayward DC, Ball EE (1997) Molecular evolution of integrins: genes encoding integrin beta subunits from a coral and a sponge. Proc Natl Acad Sci USA 94: 9182–9187. 9256456 10.1073/pnas.94.17.9182 PMC23098

[59] Wimmer W, Perovic S, Kruse M, Schroder HC, Krasko A, et al (1999) Origin of the integrin-mediated signal transduction. Functional studies with cell cultures from the sponge *Suberites domuncula* . Eur J Biochem 260: 156–165. 10091595 10.1046/j.1432-1327.1999.00146.x

[60] Burke RD (1999) Invertebrate integrins: structure, function, and evolution. Int Rev Cytol 191: 257–284. 10343395 10.1016/s0074-7696(08)60161-8

[61] Oliveira KC, Carvalho ML, Maracaja-Coutinho V, Kitajima JP, Verjovski-Almeida S (2011) Non-coding RNAs in schistosomes: an unexplored world. An Acad Bras Cienc 83: 673–694. 21670887 10.1590/s0001-37652011000200026

[62] Arnaout MA, Goodman SL, Xiong JP (2007) Structure and mechanics of integrin-based cell adhesion. Curr Opin Cell Biol 19: 495–507. 17928215 10.1016/j.ceb.2007.08.002 PMC2443699

[63] Alam N, Goel HL, Zarif MJ, Butterfield JE, Perkins HM, et al (2007) The integrin-growth factor receptor duet. J Cell Physiol 213: 649–653. 17886260 10.1002/jcp.21278

[64] Hubbard SR, Till JH (2000) Protein tyrosine kinase structure and function. Annu Rev Biochem 69: 373–398. 10966463 10.1146/annurev.biochem.69.1.373

[65] van der Flier A, Sonnenberg A (2001) Function and interactions of integrins. Cell Tissue Res 305: 285–298. 11572082 10.1007/s004410100417

[66] Ndegwa D, Krautz-Peterson G, Skelly PJ (2007) Protocols for gene silencing in schistosomes. Exp Parasitol 117: 284–291. 17870072 10.1016/j.exppara.2007.07.012 PMC2693101

[67] LaFlamme SE, Nieves B, Colello D, Reverte CG (2008) Integrins as regulators of the mitotic machinery. Curr Opin Cell Biol 20: 576–582. 18621126 10.1016/j.ceb.2008.06.006 PMC3417292

[68] Aszodi A, Hunziker EB, Brakebusch C, Fassler R (2003) Beta1 integrins regulate chondrocyte rotation, G1 progression, and cytokinesis. Genes Dev 17: 2465–2479. 14522949 10.1101/gad.277003 PMC218082

[69] Wennerberg K, Der CJ (2004) Rho-family GTPases: it's not only Rac and Rho (and I like it). J Cell Sci 117: 1301–1312. 15020670 10.1242/jcs.01118

[70] Zou W, Teitelbaum SL (2010) Integrins, growth factors, and the osteoclast cytoskeleton. Ann NY Acad Sci 1192: 27–31. 20392214 10.1111/j.1749-6632.2009.05245.x

[71] Quack T, Knobloch J, Beckmann S, Vicogne J, Dissous C, et al (2009) The formin-homology protein SmDia interacts with the Src kinase SmTK and the GTPase SmRho1 in the gonads of *Schistosoma mansoni* . PloS One 4: e6998. 19746159 10.1371/journal.pone.0006998 PMC2734992

[72] Grosse R, Copeland JW, Newsome TP, Way M, Treisman R (2003) A role for VASP in RhoA-Diaphanous signalling to actin dynamics and SRF activity. EMBO J 22: 3050–3061. 12805219 10.1093/emboj/cdg287 PMC162139

[73] Tominaga T, Sahai E, Chardin P, McCormick F, Courtneidge SA, et al (2000) Diaphanous-related formins bridge Rho GTPase and Src tyrosine kinase signaling. Mol Cell 5: 13–25. 10678165 10.1016/s1097-2765(00)80399-8

[74] Huveneers S, Danen EH (2009) Adhesion signaling - crosstalk between integrins, Src and Rho. J Cell Sci 122: 1059–1069. 19339545 10.1242/jcs.039446

[75] Lee M, Shen B, Schwarzbauer JE, Ahn J, Kwon J (2005) Connections between integrins and Rac GTPase pathways control gonad formation and function in *C. elegans* . Biochim Biophys Acta 1723: 248–255. 15716039 10.1016/j.bbagen.2005.01.003

[76] Grevelding CG (1995) The female-specific W1 sequence of the Puerto Rican strain of *Schistosoma mansoni* occurs in both genders of a Liberian strain. Mol Biochem Parasitol 71: 269–272. 7477111 10.1016/0166-6851(94)00058-u

[77] Vicogne J, Cailliau K, Tulasne D, Browaeys E, Yan YT, et al (2004) Conservation of epidermal growth factor receptor function in the human parasitic helminth *Schistosoma mansoni* . J Biol Chem 279: 37407–37414. 15231836 10.1074/jbc.M313738200

[78] Finken M, Sobek A, Symmons P, Kunz W (1994) Characterization of the complete protein disulfide isomerase gene of *Schistosoma mansoni* and identification of the tissues of its expression. Mol Biochem Parasitol 64: 135–144. 8078516 10.1016/0166-6851(94)90141-4

