# Supplementary material for: Discovery of Platyhelminth-Specific α/β-Integrin Families and Evidence for Their Role in Reproduction in Schistosoma mansoni
Source: PLoS One. 2012 Dec 27;7(12):e52519. doi: 10.1371/journal.pone.0052519 (PMC3531407; doi:10.1371/journal.pone.0052519)
Supplement: Table S1 — Protein domains of the α/β- integrin receptors from different parasitic plathyhelminths and human. Protein domains of the α- (A) and β- (B) integrin receptors from S. mansoni (Sm), S. haematobium (Sha), S. japonicum (Sjp), C. sinensis (Cs), E. multilocularis (Em), S. mediterranea (Smed), and H. sapiens (Hs) as predicted by SMART analyses and signal peptide prediction. #: NCBI accession number; *: GeneDB number, °: SmedDB number, +: SwissProt number, n.p.: not predicted. (DOCX) [file pone.0052519.s001.docx]

**Supplementary Table S1**

**Table S1 (A, B). Protein domains of the α/β- integrin receptors from different parasitic plathyhelminths and human.**

| **A: α-integrin receptors** | **length (aa)** | **signal peptide** | **Int α domains** | | **trans-membrane domain** |  |
| --- | --- | --- | --- | --- | --- | --- |
|  |  |  |  | |  |  |
| **SmαInt1** (FR749887^#^) | 1273 | 1 ‑ 39 | 255 ‑ 334, 359 ‑ 418, 449 ‑ 496  (Intα-2: 506 ‑ 877, 871 ‑ 1081) | | 1212 ‑ 1234 |  |
| **ShaαInt1** (Sha_102401^*^) | 1297 | 1 ‑ 21 | 261 ‑ 340, 365 ‑ 426, 455 ‑ 514  (Intα-2: 638 ‑ 801, 890 ‑ 1104) | | 1238 ‑ 1260 |  |
| **SjpαInt1**  (Sjp_0037690^*^, partial) | 860 | 1 ‑ 24 | 244 ‑ 319, 344 ‑ 403  (Intα-2: 590 ‑ 828) | | n.p. |  |
| **CsαInt5**  (GAA31131^#^) | 1329 | 1 ‑ 23 | 53 ‑ 121, 221 ‑ 279, 365 ‑ 470, 490 ‑ 549, 576 ‑ 623  (Intα-2: 700 ‑ 912, 950 ‑ 1200) | | 1281 ‑ 1303 |  |
| **EmαInt1**  (EmW_000215000^*^) | 1380 | 1 ‑ 39 | 113 ‑ 161, 415 ‑ 511, 535 ‑ 596, 621 ‑ 668 (Intα-2: 687 ‑ 1007, 1017 ‑ 1241) | | 1328 ‑ 1350 |  |
| **SmedαInt1**  (lcl\|mk4.001411.00.01°, partial) | 280 | n.p. | 5 ‑ 67, 76 ‑ 128, 223 ‑ 270 | | n.p. |  |
| **HsαInt5**  (NP_002196.2^#^) | 1049 | 1 ‑ 41 | 56‑ 115, 268 ‑ 318, 322 ‑ 384, 388 ‑ 444, 452 ‑ 503 | | 999 ‑ 1021 |  |
| **SmαInt2**  (FR749888^#^) | 1492 | 1 ‑ 30 | 404 ‑ 472, 476 ‑ 546, 552 ‑ 603  (Intα-2: 968 ‑ 1190) | | 1296 ‑ 1318 |  |
| **ShaαInt2**  (Sha_106921^*^, partial) | 1185 | 1 ‑ 30 | 408 ‑ 476, 480 ‑ 550, 556 ‑ 607  (Intα-2: 956 ‑ 1146) | | n.p. |  |
| **SjpαInt2**  (Sjp_0069490^*^, partial) | 1097 | n.p. | 371 ‑ 439, 443 ‑ 513, 519 ‑ 572 | | n.p. |  |
| **CsαInt-ps**  (GAA49531, GAA49530, GAA54095^#^) | 2013 | 1 ‑ 21 | 362 ‑ 422, 431 ‑ 489, 860 ‑ 923, 928 ‑ 986, 1032 ‑ 1090  (Intα-2: 1406 ‑ 1672) | | 1771 ‑ 1793 |  |
| **EmαInt2**  (EmW_000192500^*^) | 1343 | 1 ‑ 20 | 322 ‑ 378, 380 ‑ 438 | | 1150 ‑ 1172 |  |
| **SmedαInt2**  (lcl\|mk4.003797.00.01°, partial) | 397 | 1 ‑ 18 | 294 ‑ 351, 355 ‑ 396 | | n.p. |  |
| **HsαInt2b**  (EAW51595.1^#^) | 1039 | 1 ‑ 31 | 46 ‑ 103, 262 ‑ 312, 316 ‑ 377, 383 ‑ 439, 444 ‑ 495 | | 997 ‑ 1019 |  |
| **SmαInt3**  (FR749889^#^, Smp_158350^*^, Smp_156610^*^, Smp_156620^*^) | 1257 | n.p. | 178 ‑ 253, 257 ‑ 316, 345 ‑ 399, 408 ‑ 462  (Intα-2: 658 ‑ 1043) | | 1152 ‑ 1174 |  |
| **ShaαInt3**  (Sha_102914^*^) | 1259 | 1 ‑ 20 | 43 ‑ 93, 281 ‑ 340, 369 ‑ 423, 432 ‑ 495 (Intα-2: 682 ‑ 1067) | | 1154 ‑ 1176 |  |
| **SjpαInt3**  (Sjp_0063430^*^, Sjp_0063420^*^, partial) | 1080 | 1 ‑ 54 | 170 ‑ 246, 250 ‑ 309, 338 ‑ 392 | | 1055 ‑ 1077 |  |
| **CsαInt7**  (GAA52225^#^, partial) | 1242 | n.p. | 136 ‑ 186, 396 ‑ 457, 482 ‑ 536, 545 ‑ 593 (Intα-2: 770 ‑ 1180) | | n.p. |  |
| **EmαInt3**  (EmW_000782500^*^) | 1159 | n.p. | 246 ‑ 296, 333 ‑ 387, 94 ‑ 452  (Intα-2: 598 ‑ 998) | | 1077 ‑ 1099 |  |
| **SmedαInt3**  (lcl\|mk4.000046.14.01°, partial) | 295 | n.p. | 92 ‑ 141, 147 ‑ 200 | | n.p. |  |
| **HsαInt7**  (EAW96822.1^#^) | 1181 | 1 ‑ 53 | 48 ‑ 110, 303 ‑ 356, 360 ‑ 416, 421 ‑ 474, 479 ‑ 535 | | 1082 ‑ 1104 |  |
|  |  |  |  | |  |  |
| **SmαInt4**  (Smp_173540^*^, Smp_181010^*^) | 1462 | 1 ‑ 36 | 496 ‑ 550 | | 1306 ‑ 1328 |  |
| **ShaαInt4**  (Sha_104436^*^) | 1452 | n.p. | 468 ‑ 523 | | 1285 ‑ 1307 |  |
| **SjpαInt4**  (Sjp_0046780^*^, Sjp_0046790^*^) | 1184 | 1 ‑ 31 | 419 ‑ 477 | | 1049 ‑ 1071 |  |
| **CsαInt4**  (GAA28731^#^) | 1331 | 1 ‑ 19 | 359 ‑ 428, 438 ‑ 496 | | 1212 ‑ 1234 |  |
| **EmαInt4**  (EmW_000573500^*^) | 1322 | 1 ‑ 21 | 354 ‑ 408, 420 ‑ 474 | | 1166 ‑ 1188 |  |
|  |  |  |  | |  |  |
| **B: β-integrin receptors** | **length (aa)** | **signal peptide** | **Int β domain**  **(PSI, VWA)** | **EGF-like domains** | **trans-membrane domain** | |
|  |  |  |  |  |  | |
| **SmβInt1**  (FR749886^#^) | 865 | 1 ‑ 25 | 36 ‑ 470  PSI: 30 ‑ 78  VWA: 135 ‑ 392 | 567 ‑ 604  (EGF-2: 529 ‑ 561, 615 ‑ 645) | 794 ‑ 816 | |
| **ShaβInt1**  (Sha_105750^*^) | 892 | 1 ‑ 25 | 36 ‑ 467  PSI: 30 ‑ 78  VWA: 135 ‑ 392 | 594 ‑ 631  (EGF-2: 556 ‑ 588, 642 ‑ 672) | 821 ‑ 843 | |
| **SjpβInt1**  (partial, Sjp_0081260^*^) | 612 | 1 ‑ 25 | 36 ‑ 470  PSI: 30 ‑ 78  VWA: 135 ‑ 381 | n.p.  (EGF-2: 529 ‑ 561) | n.p. | |
| **CsβInt1**  (GAA31131^#^) | 825 | n.p. | 31 ‑ 431  PSI: n.d.  VWA: n.d. | 528 ‑ 565  (EGF-2: 444 ‑ 472, 490 ‑ 522, 576 ‑ 606) | 754 ‑ 776 | |
| **EmβInt1**  (EmW_000528400^*^) | 900 | 1 ‑ 23 | 32 ‑ 461  PSI: n.d.  VWA: 125 ‑ 372 | 554 ‑ 591 | 777 ‑ 799 | |
| **SmedβInt1**  (lcl\|mk4.001280.01.01°, partial) | 388 | 1 ‑ 19 | 30 ‑ 386  PSI: 24 ‑ 72  VWA: n.d. | n.p. | n.p. | |
| **HsβInt1**  (NP_000202.2^#^) | 769 | 1 ‑ 22 | 32 ‑ 447  PSI: 24 ‑ 74  VWA: 126 ‑ 357 | n.p.  (EGF-2: 582 ‑ 612) | 701 ‑ 723 | |
| **HsβInt4**  (P16144^+^) | 1822 | 1 ‑ 27 | 37 ‑ 455  PSI: 29 ‑ 73  VWA: 129 ‑ 360 | n.p.  (EGF-2: 463 ‑ 490, 543 ‑ 573) | n.p. | |

**Table I:** Protein domains of the α- (**A**) and β- (**B**) integrin receptors from *S. mansoni* (Sm)*, S. haematobium* (Sha)*, S. japonicum* (Sjp)*, C. sinensis* (Cs)*, E. multilocularis* (Em)*, S. mediterranea* (Smed)*,* and *H. sapiens* (Hs) as predicted by SMART analyses and signal peptide prediction*.* [^#^: NCBI accession number; ^*^: GeneDB number, °: SmedDB number, ^+^: SwissProt number, n.p.: not predicted]
